# Recent Progress in Thiazole, Thiosemicarbazone, and Semicarbazone Derivatives as Antiparasitic Agents Against Trypanosomatids and *Plasmodium* spp.

**DOI:** 10.3390/molecules30081788

**Published:** 2025-04-16

**Authors:** Pamela Souza Tada da Cunha, Ana Luísa Rodriguez Gini, Chung Man Chin, Jean Leandro dos Santos, Cauê Benito Scarim

**Affiliations:** Department of Drugs and Medicines, School of Pharmaceutical Sciences, Sao Paulo State University (UNESP), Araraquara 14800-903, SP, Brazil; pamela.tada@unesp.br (P.S.T.d.C.); ana.gini@unesp.br (A.L.R.G.); chung.man-chin@unesp.br (C.M.C.); jean.santos@unesp.br (J.L.d.S.)

**Keywords:** drug development, tropical diseases, structure–activity relationship (SAR), ADMET profiling

## Abstract

Neglected tropical diseases (NTDs), including Chagas disease, human African trypanosomiasis (HAT), leishmaniasis, and malaria, remain a major global health challenge, disproportionately affecting low-income populations. Current therapies for these diseases suffer from significant limitations, such as reduced efficacy, high toxicity, and emerging parasite resistance, highlighting the urgent need for new therapeutic strategies. In response, substantial efforts have been directed toward the synthesis of new molecules with improved potency, selectivity, and pharmacokinetic profiles. However, despite many of these compounds exhibiting favorable ADMET (absorption, distribution, metabolism, excretion, and toxicity) profiles and strong in vitro activity, their translation into in vivo models remains limited. Key challenges include the lack of investment, the absence of fully representative experimental models, and difficulties in extrapolating cell-based assay results to more complex biological systems. In this review, we analyzed the latest advancements (2019–2024) in the development of these compound classes, correlating predictive parameters with their observed biological activity. Among these parameters, we highlighted the partition coefficient (LogP), which measures a compound’s lipophilicity and influences its ability to cross biological membranes, and Caco-2 cell permeability, an in vitro model widely used to predict intestinal drug absorption. Additionally, we prioritized the most promising molecules and structural classes for pharmaceutical development, discussing structure–activity relationships (SARs) and the remaining challenges that must be overcome to enable the clinical application of these compounds in the treatment of NTDs.

## 1. Introduction

Neglected tropical diseases (NTDs) constitute a significant public health burden, disproportionately affecting low-income populations in tropical and subtropical regions [1,2,3]. These diseases are intrinsically linked to inadequate sanitation, limited access to potable water, and deficient sewage infrastructure, highlighting both epidemiological barriers and profound socioeconomic disparities arising from the asymmetric distribution of global resources [4,5,6,7].

In this context, initiatives such as the Drugs for Neglected Diseases Initiative (DNDi) play a pivotal role in advancing research and development efforts aimed at identifying novel therapeutic strategies [8]. However, currently available treatments often suffer from suboptimal efficacy, unfavorable toxicological profiles, and prolonged administration regimens, leading to patient non-adherence and facilitating the emergence of drug-resistant strains [9,10,11,12,13]. Consequently, medicinal chemistry has driven substantial progress in the rational design and optimization of new chemical scaffolds to yield drug candidates with enhanced safety and efficacy profiles [14,15,16].

Among the promising structural classes, thiazoles, thiosemicarbazones, and semicarbazones have garnered considerable attention due to their broad pharmacological potential [17,18,19], exhibiting anti-inflammatory [20,21], antioxidant [22,23], analgesic [24,25], antimicrobial [26,27], antitumor [28,29], anticonvulsant [30,31], neuroprotective [32,33], and antiparasitic properties [34,35]. Their synthetic accessibility, cost-effectiveness, and high structural tunability make them ideal candidates for structure–activity relationship (SAR) studies and the fine-tuning of key physicochemical parameters essential for drug discovery and development [17,36].

The thiazole nucleus is extensively explored in medicinal chemistry due to its pharmacological versatility and structural adaptability [37]. Present in over 18 FDA-approved drugs, this five-membered heterocyclic core, containing both nitrogen and sulfur atoms, facilitates diverse interactions with biological targets [17,38]. Electrophilic substitution reactions predominantly occur at C-4 and C-5, given their higher electron density, whereas nucleophilic attack is favored at C-2 following the departure of a leaving group [36,38]. The clinical significance of this scaffold is highlighted by its incorporation into widely used pharmaceuticals such as ritonavir (HIV protease inhibitor) [39,40], cefotaxime (β-lactam antibiotic) [41], meloxicam (selective cyclooxygenase-2 inhibitor) [42,43], and alpelisib (PI3Kα inhibitor for breast cancer therapy) [44,45].

Similarly, thiosemicarbazones and semicarbazones represent nitrogen-based pharmacophores with substantial medicinal relevance, primarily due to their ability to coordinate transition metals [46]. These scaffolds feature a hydrazone moiety conjugated to carbonyl or thiol groups, enabling chelation with Fe(II), Cu(II), and Zn(II) [46,47]. This metal-binding capability modulates critical physicochemical properties, such as lipophilicity, bioavailability, and chemical stability, often enhancing cellular uptake and conferring novel biological activities while mitigating drug resistance [47,48]. Mechanistically, these compounds exert their pharmacological effects via ribonucleotide reductase (RR) inhibition, a key enzyme in deoxyribonucleic acid (DNA) biosynthesis, and generation of reactive oxygen species (ROS), inducing oxidative stress-mediated cytotoxicity against tumor cells and pathogenic microorganisms [19,49,50].

**Figure 1:**
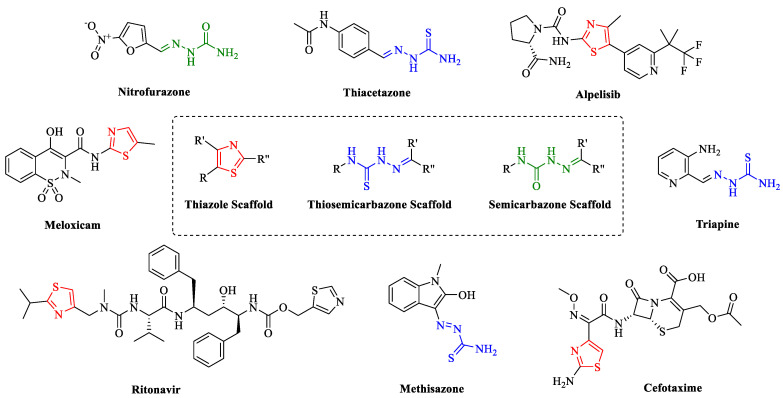
Approved drugs containing thiazole, thiosemicarbazone, and semicarbazone scaffolds.

The therapeutic significance of these compounds is exemplified by triapine (3-AP), a potent RR inhibitor under clinical investigation for cancer treatment [51,52]; Thiacetazone, an antimycobacterial agent used in multidrug-resistant tuberculosis therapy [53,54]; methisazone, an antiviral agent that inhibits mRNA and protein synthesis [55,56]; and nitrofurazone, applied for the prevention and treatment of wound, burn, and ulcer infections [57] (Figure 1). Ongoing SAR-driven optimization continues to yield novel derivatives with improved selectivity and reduced toxicity, expanding their therapeutic applications [58,59].

Beyond these main classes, other sulfur- and nitrogen-containing analogs, such as thiadiazoles and thiazolidinones, have also been explored as promising antiparasitic agents, particularly for targeting essential metabolic pathways in pathogenic protozoa [60,61]. Notably, their incorporation into drug design strategies has yielded promising candidates targeting essential protozoan metabolic processes [62]. These compound classes share electronic and pharmacophoric features that enable specific interactions with biological targets while providing structural flexibility for the optimization of pharmacokinetic and pharmacodynamic properties [63].

In 2019, Scarim and colleagues provided a comprehensive review on the medicinal chemistry of thiazoles, thiosemicarbazones, and semicarbazones [64]. Expanding on this foundation, the present review aims to update the current knowledge on these structural scaffolds by examining recent advancements and rational drug design strategies that have propelled the discovery of promising drug candidates. Special emphasis is placed on the optimization of pharmacokinetic and pharmacodynamic properties to enhance their clinical translation potential.

This review also provides a critical analysis of recent studies (2019–2024) exploring the application of these compounds in the treatment of Chagas disease, human African trypanosomiasis (HAT), leishmaniasis, and malaria. Literature searches conducted across PubMed, ScienceDirect, Scopus, and Web of Science offer an updated perspective on their therapeutic potential in medicinal chemistry, highlighting the latest advances and challenges in this field.

### 1.1. Chagas Disease

American trypanosomiasis, or Chagas disease, is an NTD caused by the flagellated protozoan *Trypanosoma cruzi* (*T. cruzi*) and primarily transmitted by more than 140 species of insect vectors belonging to the Triatominae subfamily [65,66,67]. Although the disease is endemic in 21 countries across the Americas, globalization and factors such as the migration of infected individuals [68,69], contaminated blood transfusions [70,71], accidental ingestion of the vector [72,73], and laboratory accidents [74,75] have facilitated its spread. As a result, the global prevalence is estimated to range between 6 and 8 million cases [76,77]. Additionally, approximately 75 million people live at risk of infection, with an annual incidence of 30,000 to 40,000 new cases and a mortality rate that can reach up to 12,000 deaths per year [78,79].

Currently, the available treatment is limited to benznidazole (BZN) and nifurtimox, both of which were developed over five decades ago and exhibit limited efficacy, primarily restricted to the acute phase of the disease [80,81,82]. Delayed diagnosis and the adverse effects associated with these drugs significantly reduce their clinical applicability, contributing to disease progression [83,84,85]. Approximately 30% of patients develop the chronic form, often leading to severe cardiomyopathy and irreversible heart failure [86]. Given these limitations, the search for new chemical entities with improved pharmacological profiles and enhanced efficacy against *T. cruzi* has become a major focus in medicinal chemistry.

Among the most promising molecular frameworks, heterocyclic scaffolds such as thiazole, thiosemicarbazone, and semicarbazone derivatives have been extensively explored due to their versatile pharmacological properties and their ability to interact with key biological targets in *T. cruzi*. Several studies have reported significant antiparasitic activity within these structural classes, supporting their potential as new drug candidates for Chagas disease [87,88].

**Figure 2:**
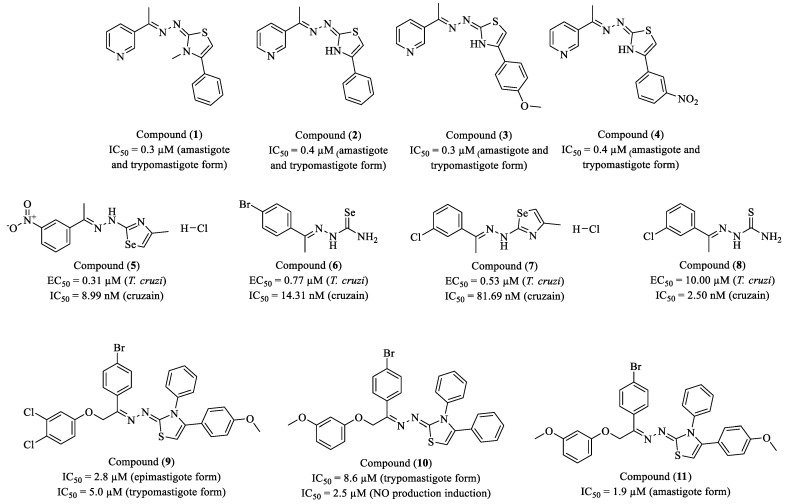
Representative structures of thiazole, thiosemicarbazone, and semicarbazone derivatives (compounds **1**–**11**) with antiparasitic activity against *T. cruzi* and cruzain. The IC_50_ and EC_50_ values highlight their potency, providing insights into structure–activity relationships and potential for further optimization.

Silva et al. (2024) investigated a new series of 18 compounds containing the 3-pyridyl-1,3-thiazole subunit as potential agents against diseases caused by Trypanosomatidae parasites, including Chagas disease (*T. cruzi*) and leishmaniasis (*Leishmania* spp.) [89]. Among the compounds, 14 derivatives exhibited trypanocidal activity with half maximal inhibitory concentration (IC_50_) values ranging from 0.2 to 3.9 μM, showing greater potency than BZN (IC_50_ = 4.2 μM). Compounds (**1**), (**2**), (**3**), and (**4**) displayed a trypanostatic effect, promoting mitochondrial disruption, apoptosis, and parasite membrane damage, as well as immunomodulatory effects, including increased nitric oxide (NO) and IL-6 production and decreased IL-10 levels in macrophages (Figure 2). Compounds (**2**) (IC_50_ = 0.4 μM, SI = 530.8) and (**4**) (IC_50_ = 0.4 μM, SI = 284.2) stood out for their high selectivity. In silico analysis indicated strong interactions with cruzain and pharmacokinetic properties compatible with oral drugs. However, these compounds were ineffective in murine models after oral administration (100 mg/kg/day), suggesting challenges related to stability and bioavailability. The compounds showed negligible or no activity against *L. amazonensis* and *L. infantum*. Thus, despite their promising in vitro activity against *T. cruzi*, the lack of in vivo efficacy highlights the need for structural optimization and formulation strategies to enhance their therapeutic potential.

Rubio-Hernández and co-workers (2024) synthesized a series of 57 novel sulfur (S)- and selenium (Se)-containing compounds, including thiosemicarbazones, thiazoles, selenosemicarbazones, and selenazoles, to evaluate their activity against *T. cruzi* and cruzain [90]. The study employed a bioisosteric replacement strategy, substituting sulfur with selenium to enhance antiparasitic activity and reduce toxicity. Biological screening identified four promising compounds: (**5**), (**6**), (**7**), and (**8**) (Figure 2). Among these, compound (**5**) exhibited the highest potency, with EC_50_ values of 0.31 μM against *T. cruzi* and 8.99 nM (0.00899 µM) against cruzain, along with an SI of 148.83, indicating high specificity for the enzymatic target. Structural characterization and molecular dynamics simulations suggested a non-covalent binding mode to cruzain, featuring hydrogen bonds with Leu160 and Gly66, as well as CH-π and π-hole interactions, which stabilize the enzyme–ligand complex. Pharmacokinetic studies revealed an oral bioavailability of 82%, although the compound displayed a short half-life (<1 h), indicating the need for further optimization before in vivo evaluation. Compound (**6**) demonstrated an EC_50_ of 0.77 μM against *T. cruzi* and an IC_50_ of 14.31 nM (0.01431 μM) against cruzain, with an SI of 7.42, indicating moderate antiparasitic activity. Compound (**7**) exhibited an EC_50_ of 0.53 μM and an IC_50_ of 81.69 nM (0.08169 μM), with an SI of 5.55, suggesting moderate efficacy yet lower selectivity. Conversely, compound (**8**) showed strong cruzain inhibition (IC_50_ = 2.50 nM; 0.0025 μM) while displaying a high EC_50_ of 10 μM against *T. cruzi*, indicating its primary activity as an enzyme inhibitor without a direct impact on parasite viability. SAR analysis revealed that incorporating selenium into cyclic structures (selenazoles, SeZ) enhanced antiparasitic activity while reducing toxicity, making this an attractive strategy for the development of selective cruzain inhibitors. Compound (**5**) emerges as a promising lead compound, warranting further optimization to improve its pharmacokinetic properties and advance toward in vivo evaluation for Chagas disease treatment.

Cristovão-Silva et al. (2024) reported the synthesis and characterization of 13 new phenoxy-hydrazino-thiazole derivatives with potential activity against *T. cruzi* [91]. Among the compounds evaluated, (**9**), (**10**), and (**11**) exhibited the most promising biological profiles, standing out for their potency and selectivity (Figure 2). Compound (**9**) demonstrated an IC_50_ value of 2.8 μM against epimastigotes and 5 μM against trypomastigotes. Additionally, it stimulated NO production (200 μg/mL) and showed affinity for TLR2 and TLR4 receptors, suggesting a potential immunomodulatory effect. Regarding cytotoxicity, it exhibited moderate toxicity toward mammalian cells (CC_50_ between 58.1 and 100.3 μM), while displaying high selectivity for epimastigotes, being up to 50 times more selective for the parasite than for fibroblasts. The compound (**10**) exhibited an IC_50_ of 8.6 μM against trypomastigotes and an IC_50_ value of 2.5 μM for NO production induction. Molecular modeling predicted affinity for TNFR, IFNGR, and IL-2R, indicating potential modulation of the host immune response. Furthermore, (**10**) induced apoptosis in parasites, reduced mitochondrial membrane potential, and promoted the reduction in acidic compartments within the parasite, suggesting an effect on lysosomal homeostasis. Immunologically, (**10**) stimulated the production of TNF-α, IFN-γ, IL-2, IL-4, IL-10, and IL-17, highlighting a broad immunomodulatory profile. Regarding toxicity, it exhibited CC_50_ values between 30.6 and 96.6 μM in mammalian cells while maintaining high selectivity for trypomastigotes, with an SI up to 19 times higher for the parasite than for macrophages. Compound (**11**) stood out with an IC_50_ of 1.9 μM against amastigotes, making it the most potent among the three derivatives. Its chemical structure features a methoxy group at the terminal position of the benzene ring, which increases its lipophilicity compared to (**10**), possibly enhancing its penetration into host cells. In terms of immunomodulation, it induced TNF-α and IL-6 production, activating a pro-inflammatory response without IL-4 or IL-10 induction, which may be beneficial for parasite clearance. Regarding toxicity, it exhibited low toxicity toward mammalian cells (CC_50_ > 100 μM in some cell lines). Notably, (**11**) displayed exceptional selectivity for amastigotes, being 117 times more selective for the parasite than for hepatic cells, making it a promising candidate for further studies.

**Figure 3:**
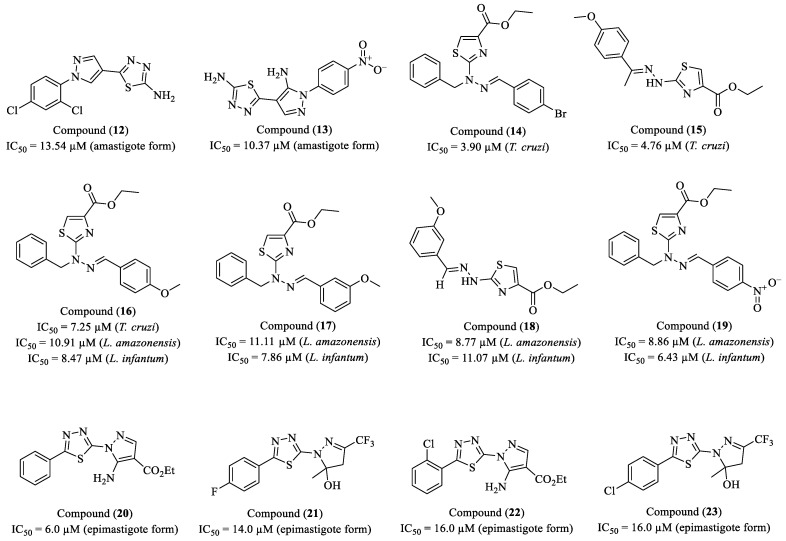
Chemical structures of thiazole, thiosemicarbazone, and semicarbazone derivatives (compounds **12**–**23**) with antiparasitic activity against *T. cruzi* and *Leishmania* spp. The IC_50_ values highlight their potency against different parasite forms, with key structural variations influencing bioactivity. Substituents such as halogens, nitro, and methoxy groups modulate interactions with biological targets, impacting selectivity and efficacy.

Souza and researchers (2024) synthesized and evaluated two series of pyrazole–thiadiazole derivatives for their antiparasitic activity against the intracellular and trypomastigote forms of *T. cruzi* [92]. The most active compounds were (**12**) and (**13**), with IC_50_ values of 13.54 µM (SI > 32.96) and 10.37 µM (SI > 33.87), respectively, against intracellular amastigotes (Figure 3). Compound (**12**) also exhibited activity against trypomastigotes, with an IC_50_ of 21.71 µM (SI > 23.03). Ultrastructural analysis revealed that (**12**) induced flagellar detachment from the parasite body, suggesting a possible mechanism of action involving structural proteins of the flagellum and the Flagellar Attachment Zone. In 3D cardiac microtissues infected with *T. cruzi*, compound (**13**) reduced the parasite load by up to 86% at 100 µM, while (**12**) decreased infection by 57% at the same concentration. The effect of (**13**) was comparable to that of BZN. In infection recrudescence (washout) assays, (**13**) reduced trypomastigote release by up to 93% yet failed to prevent infection relapse, indicating a trypanostatic effect. The combination of (**12**) and (**13**) with BZN resulted in an additive effect (ΣFICI ≈ 1.02–1.12), suggesting that these compounds could be explored in combination therapy to optimize Chagas disease treatment.

Haroon et al. (2024) synthesized 28 novel 1,3-thiazole-4-carboxylate derivatives and their *N*-benzylated analogs, evaluating their in vitro activity against intracellular amastigote forms of *T. cruzi* and both promastigote and intracellular amastigote forms of *L. infantum* and *L. amazonensis* [93]. Among the synthesized compounds, six exhibited significant antiparasitic activity, namely (**14**), (**15**), (**16**), (**17**), (**18**), and (**19**) (Figure 3). Compound (**14**) demonstrated an IC_50_ of 3.90 μM (SI = 15.48) against *T. cruzi*, exhibiting greater potency than BZN (IC_50_ = 4.82 μM). Compounds (**15**) and (**16**) also displayed potent trypanocidal activity, with IC_50_ values of 4.76 μM (SI = 14.56) and 7.25 μM (SI = 28.98), respectively. Compound (**16**) also showed prominent leishmanicidal activity, particularly against promastigotes of *L. amazonensis* (IC_50_ = 10.91 μM, SI = 19.26) and *L. infantum* (IC_50_ = 8.47 μM, SI = 24.81). Compounds (**17**), (**18**), and (**19**) were also active against both leishmania species. Compound (**18**) exhibited an IC_50_ of 8.77 μM (SI = 13.17) against *L. amazonensis* and 11.07 μM (SI = 10.43) against *L. infantum*. Compound (**19**) was effective against *L. amazonensis* (IC_50_ = 8.86 μM, SI = 6.43) and *L. infantum* (IC_50_ = 6.23 μM, SI = 9.15). Additionally, compound (**17**) exhibited IC_50_ values of 11.11 μM (SI = 10.65) and 7.86 μM (SI = 15.05) for *L. amazonensis* and *L. infantum*, respectively. The SAR analysis indicated that the presence of methoxy (-OCH_3_) and bromo (-Br) groups enhanced antiparasitic activity, with (**14**), containing a bromine substituent, emerging as the most potent compound against *T. cruzi*. *N*-benzylation also positively influenced biological activity, likely due to increased lipophilicity and improved interaction with the target. Additionally, the introduction of nitro (-NO_2_) groups contributed to leishmanicidal activity, although with lower selectivity.

Faria and colleagues (2023) investigated the biological activity of a series of previously synthesized 2-(1H-pyrazol-1-yl)-1,3,4-thiadiazole derivatives, aiming to assess their potential against *T. cruzi* [94]. Among the evaluated compounds, four exhibited the highest efficacy against the epimastigote form of the parasite: (**20**), with an IC_50_ of 6.0 µM; (**21**), with an IC_50_ of 14.0 µM; (**22**), with an IC_50_ of 16.0 µM; and (**23**), with an IC_50_ of 18.0 µM (Figure 3). Cytotoxicity assays demonstrated that all compounds exhibited low toxicity in murine peritoneal macrophages (CC_50_ > 100 µM), suggesting a promising selective profile. From a mechanistic perspective, (**20**) and (**22**) were the most potent inducers of ROS. The ethidium bromide uptake assay suggested that these compounds may compromise the integrity of the parasite’s cell membrane. Notably, (**23**) displayed the most significant trypanocidal effect against trypomastigote forms, effectively reducing parasite viability across all tested concentrations. The in silico pharmacokinetic assessment indicated that these compounds possess favorable intestinal absorption, moderate systemic distribution, and metabolism predominantly mediated by CYP1A2 and CYP3A4, suggesting the potential for metabolic interactions [95].

**Figure 4:**
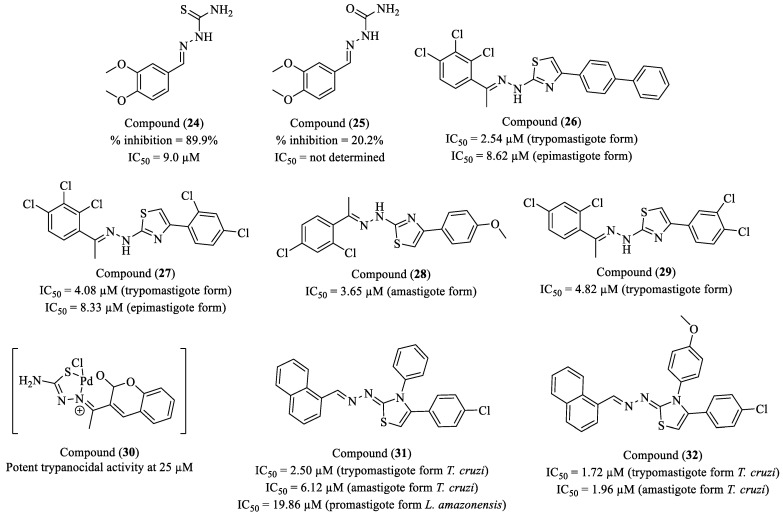
Chemical structures of selected thiazole, thiosemicarbazone, and semicarbazone derivatives (compounds **24**–**32**) evaluated for their trypanocidal activity. Structural modifications, including electron-withdrawing and electron-donating groups, influence bioactivity, while heterocyclic scaffolds modulate selectivity and target binding affinity.

Martins and collaborators (2023) investigated the mechanism of cruzain inhibition by two thiosemicarbazones using a combination of computational and experimental approaches [96]. Compound (**24**) exhibited 89.9% inhibition at 100 µM, with an IC_50_ of 9.0 µM, whereas compound (**25**) showed low activity, achieving only 20.2% inhibition at the same concentration, with no IC_50_ determination (Figure 4). Enzymatic assays confirmed that compound (**24**) acts as a reversible covalent inhibitor, following a two-step inhibition mechanism, with a ΔG of −1.4 kcal/mol, indicating a favorable equilibrium between the pre-covalent and covalent states. Molecular docking studies revealed that compound (**24**) establishes key interactions with Gly66, Leu67, Glu208, and Ala138, residues located in the S2 subsite of cruzain. The stabilization of the pre-covalent complex was strongly influenced by the positioning of the dimethoxyphenyl group within this subsite, ensuring the anchoring of the thiosemicarbazone in the catalytic region. Molecular dynamics simulations further demonstrated that the thiosemicarbazone moiety remains rigidly positioned, whereas the aromatic group exhibits greater flexibility. Conversely, compound (**25**) displayed greater structural flexibility and lower stability in the active site, which may explain its reduced inhibitory activity. These findings suggest that differences in the stability of the pre-covalent complex between the two compounds are directly correlated with their efficacy as cruzain inhibitors.

Dias and colleagues (2023) conducted the rational design, synthesis, and in silico studies of a series of 16 novel 1,3-thiazole derivatives, exploring various patterns of halogenated and phenolic substitution [97]. The activity of these compounds was assessed against the epimastigote, amastigote, and trypomastigote forms of *T. cruzi*. Furthermore, mechanistic studies were performed, suggesting that the compounds induce apoptosis in the parasites without compromising mitochondrial membrane potential, a relevant distinction for minimizing toxic effects on host cells. Among the tested derivatives, compounds (**26**), (**27**), (**28**), and (**29**) exhibited the most promising activity (Figure 4). Compound (**26**) displayed the highest potency against the trypomastigote form, with an IC_50_ of 2.64 μM, an SI of 23.57, and was 24-fold more selective than BZN. Against the epimastigote form, it showed an IC_50_ of 8.62 μM (SI = 7.22). Compound (**27**) demonstrated notable efficacy against both the trypomastigote (IC_50_ = 4.08 μM, SI = 8.89, 9-fold more selective than BZN) and epimastigote forms (IC_50_ = 8.33 μM, SI = 4.35). Compound (**28**) exhibited a marked effect against amastigotes, with an IC_50_ of 3.65 μM and an SI of 11.82, being 12-fold more selective than BZN. Compound (**29**) showed potent activity against trypomastigotes (IC_50_ = 4.82 μM, SI = 20.18), demonstrating 20-fold higher selectivity than BZN. Molecular modeling studies suggested that these compounds interact with cruzain, potentially contributing to their mechanism of action. Furthermore, an evaluation of physicochemical and pharmacokinetic properties indicated that all compounds comply with Lipinski’s and Veber’s rules, suggesting a favorable drug-like profile. Notably, compound (**29**) exhibited high selectivity and induced significant DNA damage in the parasite, being 5-fold more potent than BZN in this regard, which may enhance its antiparasitic efficacy.

Rostán and colleagues (2023) synthesized and characterized two new Pd(II) and Pt(II) metal complexes incorporating the coumarin–thiosemicarbazone hybrid HL1 as a ligand, aiming to optimize antiparasitic activity through structural modifications. The biological evaluation of these compounds demonstrated significant efficacy against *T. cruzi*, targeting both extracellular (trypomastigote) and intracellular (amastigote) forms, as well as inhibiting parasite release from infected macrophages [98]. Among the tested compounds, (**30**) exhibited the most promising profile, displaying a potent trypanocidal effect in vitro at 25 µM, completely eliminating trypomastigotes within 24 h (Figure 4). Moreover, the compound significantly reduced the intracellular amastigote burden by ~60% at 12 µM, demonstrating strong inhibition of parasite replication within macrophages. It also impaired trypomastigote release, leading to an ~80% reduction in parasite counts on day 5 post-infection, suggesting a potential impact on disease progression. The selectivity profile of (**30**) was favorable, as it effectively targeted the parasite with minimal toxicity to macrophages at therapeutic concentrations. Cell viability assays revealed only mild cytotoxicity at concentrations exceeding 50 µM, supporting its safety profile. In in vivo studies, a 1 mg/kg dose significantly reduced parasitemia without evident toxicity, reinforcing the potential of this complex as a promising lead for antichagasic drug development.

Filho and colleagues (2023) investigated the biological potential of eight 4-(4-chlorophenyl) thiazole derivatives, evaluating their pharmacokinetic properties, antioxidant activity, cytotoxicity in mammalian cells, and antiparasitic activity against *L. amazonensis* and *T. cruzi* [99]. Among the tested compounds, (**31**) and (**32**) exhibited the best activity profiles (Figure 4). For *T. cruzi*, (**31**) and (**32**) showed IC_50_ = 2.50 µM and 1.72 µM against the trypomastigote form (SI = 80 and 116, respectively) and IC_50_ = 6.12 µM and 1.96 µM against the amastigote form (SI = 32.68 and 102.04). For *L. amazonensis*, (**31**) demonstrated the highest efficacy against the promastigote form (IC_50_ = 19.86 µM, SI = 10.07). In silico analyses revealed high intestinal permeability (>95%) yet low aqueous solubility (LogS < −8.0), suggesting challenges in formulation. Both compounds exhibited high lipophilicity (LogP > 5), which may favor tissue distribution while potentially impacting absorption and metabolism. Moreover, antioxidant assays indicated low activity, suggesting that the antiparasitic effect is not related to this mechanism. Despite their promising antiparasitic potential, structural optimizations are required to enhance their solubility and bioavailability, aiming for further development as drug candidates against *T. cruzi* and *L. amazonensis*.

**Figure 5:**
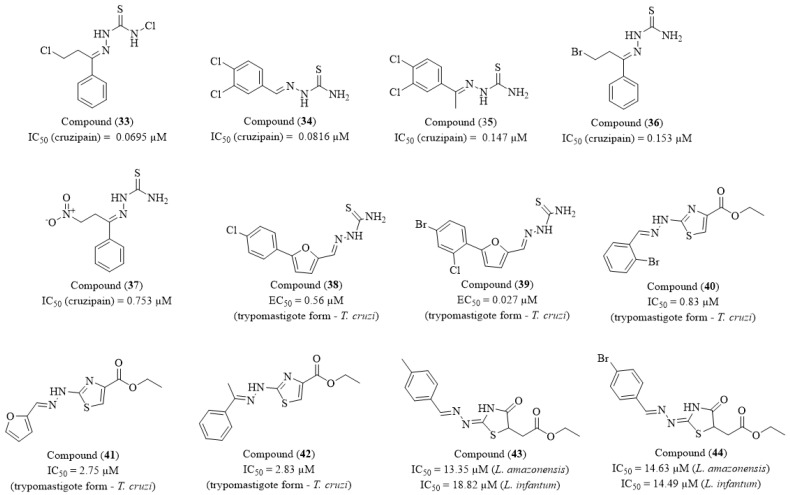
Representative thiazole, thiosemicarbazone, and semicarbazone derivatives (compounds **33**–**44**) evaluated for their trypanocidal activity. Structural variations, including heterocyclic frameworks and electronic substituents, influence inhibitory potency against *T. cruzi*, cruzain and *Leishmania*.

Jasinski and co-workers (2022) synthesized and characterized a series of 11 thiosemicarbazone derivatives and evaluated their inhibitory activity against cruzipain [100]. Additionally, molecular docking studies were conducted to elucidate the binding interactions, and an SAR analysis was established. Among the synthesized compounds, five exhibited the most potent inhibitory activity: compounds (**33**), (**34**), (**35**), (**36**), and (**37**) (Figure 5). Compound (**33**) displayed an IC_50_ of 0.0695 µM, followed by compound (**34**) with an IC_50_ of 0.0816 µM. Compound (**35**) demonstrated comparable potency with an IC_50_ of 0.147 µM, whereas compound (**36**) exhibited an IC_50_ of 0.153 µM. Lastly, compound (**37**) showed an IC_50_ of 0.753 µM, indicating a lower, yet still significant, inhibitory effect within the evaluated series. Molecular docking studies revealed that the most potent inhibitors adopt a favorable orientation within the cruzipain active site, establishing key interactions with Cys25 and His162, which are critical for enzymatic activity. The presence of dichlorinated substituents in compounds (**33**), (**34**), and (**35**) enhanced binding affinity and stabilization of the enzyme–ligand complexes, while the brominated analogue (**36**) exhibited a comparable effect. In contrast, compound (**37**), bearing a nitro substituent, displayed reduced potency, suggesting suboptimal binding interactions. These findings underscore the significant role of aromatic substitution patterns in modulating cruzipain inhibition, reinforcing the importance of precise structural modifications to optimize enzyme–ligand interactions.

Braga et al. (2022) designed and synthesized new compounds derived from a previously identified inhibitor and conducted both enzymatic and in vitro assays [101]. The thiosemicarbazones (**38**) and (**39**), based on a furan scaffold, exhibited potent activity (IC_50_ = 0.04 µM and IC_50_ = 0.05 µM, respectively) against the *Tb*CatL (*Trypanosoma brucei* cathepsin L) enzyme, initially suggesting a covalent inhibition mechanism (Figure 5). However, further analysis revealed that the IC_50_ values varied significantly in the absence of surfactants such as Triton X-100 and in the presence of BSA, indicating a behavior characteristic of colloidal aggregators. This finding suggests that the observed inhibition may not be solely due to a specific interaction with the target enzyme; instead, it could be an artifact caused by aggregate formation. Despite their colloidal aggregation behavior, these thiosemicarbazones exhibited significant trypanocidal activity in vitro, with EC_50_ values of 0.56 µM and 27 nM against *T. cruzi* trypomastigotes, surpassing BZN 200-fold. Notably, compound (**39**) displayed an exceptionally high SI (SI > 370), making it a promising candidate for further optimization and in vivo evaluation.

Haroon and researchers (2021) designed, synthesized, and evaluated a series of 27 new 1,3-thiazole and 4-thiazolidinone derivatives for their potential trypanocidal and leishmanicidal effects [102]. Given the challenges associated with Chagas disease, the authors focused on assessing activity against *T. cruzi*, where 1,3-thiazole derivatives exhibited the most promising results. Compound (**40**) displayed an IC_50_ of 0.83 µM and an SI of 303.2, suggesting a favorable balance between potency and selectivity. Other derivatives also demonstrated significant activity, including (**41**) with an IC_50_ of 2.75 µM and an SI of 153.6, and (**42**), which exhibited an IC_50_ of 2.83 µM and an SI of 103.3, reinforcing the relevance of this scaffold as a potential starting point for new Chagas disease therapies. For leishmaniasis, the 4-thiazolidinone derivatives exhibited the highest activity. Compound (**43**) showed an IC_50_ of 13.35 µM against *L. amazonensis* with an SI of 45.7, while against *L. infantum*, it presented an IC_50_ of 18.82 µM and an SI of 32.4. Similarly, (**44**) demonstrated IC_50_ values of 14.63 µM against *L. amazonensis* (SI = 27.2) and 14.49 µM against *L. infantum* (SI = 27.4), highlighting the potential of this scaffold for leishmanicidal drug development (Figure 5). Beyond biological characterization, the authors investigated the mechanism of cell death induced by (**40**) in *T. cruzi*. Flow cytometry analysis revealed that this derivative induces both necrosis and apoptosis, which may contribute to its efficacy. Additionally, scanning electron microscopy (SEM) showed notable morphological alterations in the parasite, such as flagellum shortening, cell body deformations, and cytoplasmic content leakage, suggesting a direct effect on the protozoan’s structure.

**Figure 6:**
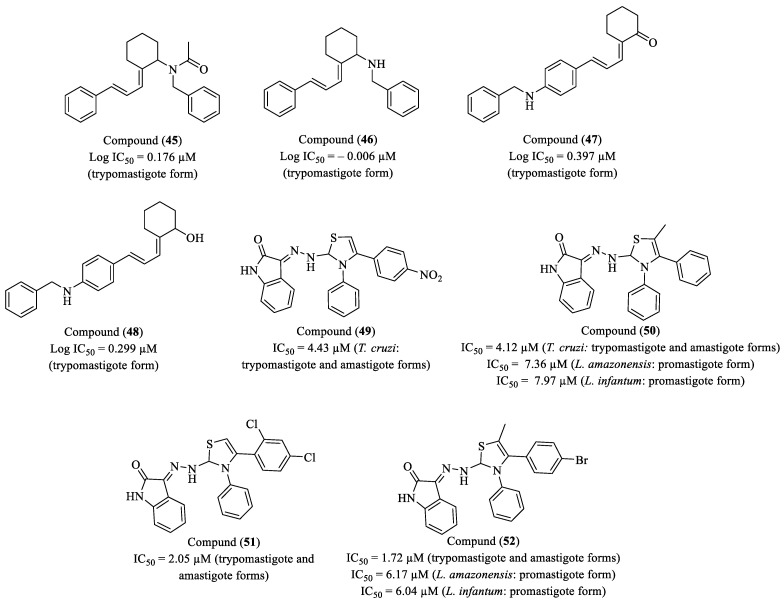
Structures of selected bioactive thiazole, thiosemicarbazone, and semicarbazone derivatives (compounds **45**–**52**) evaluated for their trypanocidal potential. IC_50_ values and logarithmic potency data illustrate their activity against *T. cruzi* in different parasite stages. Structural modifications, including aromatic substitutions and heterocyclic frameworks, influence bioactivity and selectivity, providing valuable insights for further lead optimization.

González and researchers (2021) applied in silico quantitative structure–activity relationship (QSAR) models to predict the antiprotozoal activity of thiosemicarbazone and thiazole derivatives against *T. cruzi*, based on biological activity data previously reported in the literature [103,104]. Among the compounds evaluated, molecules (**45**), (**46**), (**47**), and (**48**) were identified as the most promising candidates for *T. cruzi* treatment, selected based on three key factors: antiparasitic potency, host cell toxicity, and SI (Figure 6). Molecule (**46**) showed a log IC_50_ of 0.176 µM, reflecting potent antiparasitic activity, with a log CC_50_ of 0.885 µM, suggesting a relatively low toxicity profile for host cells. The log SI of 0.709 indicates a favorable balance between antiparasitic activity and host safety. Molecule (**46**), in turn, exhibited a log IC_50_ of −0.006 µM and a log CC_50_ of 1.158 µM, standing out for its excellent selectivity with a log SI of 1.164, suggesting a robust therapeutic profile and low toxicity risk. Molecule (**47**), with a log IC_50_ of 0.397 µM and log CC_50_ of 0.959 mM, displayed a log SI of 0.562, indicating an acceptable trade-off between efficacy and toxicity, although with slightly lower selectivity compared to the others. Molecule (**48**), with a log IC_50_ of 0.299 µM and log CC_50_ of 0.925 µM, yielded a log SI of 0.626, reinforcing its therapeutic potential. When compared to BZN, which has a log IC_50_ and log CC_50_ of 0.792 µM and a log SI of 1.668, molecules (**45**), (**46**), and (**48**) show equivalent or superior performance, with the added benefit of a more favorable toxicity profile, except for molecule (**47**), which has slightly lower selectivity [105].

Freitas et al. (2021) synthesized and evaluated thiazolyl-isatin derivatives for their activity against *T. cruzi* trypomastigote forms and *L. infantum* and *L. amazonensis* promastigote forms [106]. The compounds were obtained from thiosemicarbazones and subjected to cytotoxicity studies in RAW 264.7 macrophages. In vitro assays revealed that compounds (**49**) (IC_50_ = 4.43 µM), (**50**) (IC_50_ = 4.12 µM), (**51**) (IC_50_ = 2.05 µM), and (**52**) (IC_50_ = 1.72 µM) exhibited strong activity against *T. cruzi* trypomastigotes (Figure 6). Among these, compound (**52**) was the most potent, being eight times more active than BZN (IC_50_ = 14.60 µM). The SAR analysis indicated that the presence of a phenyl group at the N3 position of the thiazoline enhanced antiparasitic activity, making these compounds more potent than thiosemicarbazone derivatives. For leishmania, compounds (**50**) (IC_50_ = 7.36 µM for *L. amazonensis* and 7.97 µM for *L. infantum*) and (**52**) (IC_50_ = 6.17 µM and 6.04 µM, respectively) displayed the highest activity and demonstrated a higher SI than miltefosine, suggesting a more favorable toxicity profile for further studies. Mechanistic studies indicated that compound (**52**) induced structural deformations in *T. cruzi* trypomastigotes, causing flagellar alterations and cytoplasmic leakage, as observed by SEM. Flow cytometry analysis demonstrated that (**50**), (**51**), and (**52**) induce necrotic cell death in parasites. Additionally, X-ray crystallography revealed structural differences between series thiosemicarbazone and thiazoline, with the latter exhibiting a distinct configuration due to hydrogen interactions and electronic rearrangements, which may explain their enhanced biological potency. The compounds exhibited low toxicity to RAW 264.7 macrophages (CC_50_ > 45 µM), and in silico analyses indicated compliance with Lipinski’s rule of five, as well as good gastrointestinal absorption.

**Figure 7:**
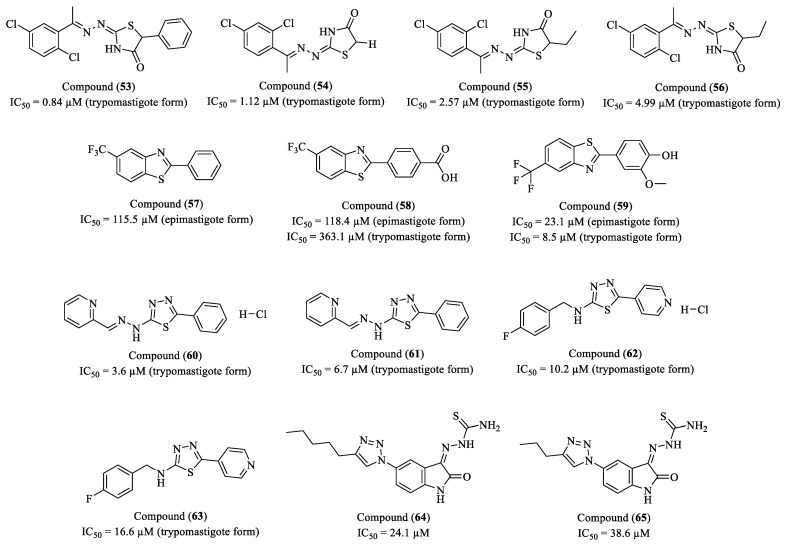
Structures of thiazole, thiosemicarbazone, and semicarbazone derivatives (compounds **53**–**65**) with trypanocidal activity. IC_50_ values highlight the influence of halogenated and fluorinated substituents on bioactivity and selectivity against *T. cruzi*.

Filho and colleagues (2021) synthesized and evaluated a novel series of 4-thiazolidinone derivatives for their in vitro antiparasitic activity against *T. cruzi* [107]. Among the synthesized compounds, four exhibited the most promising activities: (**53**), (**54**), (**55**), and (**56**) (Figure 7). Compound (**53**) demonstrated an IC_50_ of 0.84 µM against trypomastigotes (SI = 15.79) and induced both necrosis and apoptosis in the parasites. Compound (**54**) exhibited an IC_50_ of 1.12 µM, displaying the highest SI (SI = 74.16) among all tested compounds—approximately five times more selective than BZN (SI = 15.34). Additionally, (**54**) significantly reduced the release of trypomastigotes from infected macrophages without inducing cytotoxicity. Compound (**55**) presented an IC_50_ of 2.57 µM (SI = 11.82) and caused severe morphological alterations in the parasite, including flagellar shortening and cytoplasmic content leakage. Meanwhile, compound (**56**) exhibited an IC_50_ of 4.99 µM and stood out due to its low cytotoxicity in splenocytes (lethal dose 50%, LD_50_ = 151.97 µM, SI = 30.45) and its ability to reduce the release of trypomastigotes from infected macrophages.

Martínez-Cerón and co-workers (2021) investigated the in vitro and in vivo trypanocidal properties of four 2-phenylbenzothiazole derivatives against *T. cruzi* [108]. Among these, (**57**) and (**58**) emerged as the most effective, with IC_50_ values of 115.5 μM and 118.4 μM against epimastigotes, respectively (Figure 7). Regarding trypomastigotes, (**58**) exhibited an IC_50_ of 363.1 μM, displaying greater potency than BZN (IC_50_ = 1229 μM). The SI for epimastigotes was determined as 10.4 for (**57**) and 10.1 for (**58**), while (**58**) showed an SI of 3.3 against trypomastigotes, suggesting a more favorable selectivity profile than BZN (SI = 0.42). In in vivo evaluations, (**58**) reduced parasitemia by 40% within the first six hours following a single administration. However, its long-term efficacy was restricted, with a maximum parasitemia reduction of 30% observed on day 20 at 16 mg/kg, while other doses failed to produce a significant effect. Toxicity assessments revealed that (**58**) did not cause hepatotoxicity under repeated administration. However, at higher doses (>800 mg/kg), it induced neurological effects such as seizures and weight loss, suggesting CNS involvement. Additionally, (**58**) altered the protein profile of neuronal cells, raising concerns about potential neurotoxicity. Although (**58**) demonstrated strong trypanocidal activity in vitro, its limited in vivo efficacy underscores the need for structural optimization to enhance its therapeutic potential and minimize possible adverse effects.

Cuevas-Hernández et al. (2020) reported the synthesis, structural characterization, and biological evaluation of 14 benzothiazole derivatives with potential activity against *T. cruzi* [109]. Among the compounds investigated, (**59**) exhibited the highest efficacy, with an IC_50_ of 23.1 µM for epimastigotes and 8.5 µM for trypomastigotes, in addition to reducing CHO-K1 cell infection by 81.3%. The presence of hydroxyl (-OH) and methoxy (-OCH_3_) groups on the phenyl ring may enhance its biopharmaceutical profile and molecular interactions, favoring permeability and target engagement (Figure 7). Mechanistic studies indicated that (**59**) induces cell cycle arrest at the G2/M phase, likely due to interference with DNA replication. Furthermore, TUNEL assays demonstrated a marked selectivity for kinetoplast DNA (68%), with minimal impact on nuclear DNA (5%), suggesting a preferential effect on the kinetoplast. The compound also induced changes in mitochondrial membrane potential (Δψm) and intracellular Ca^2+^ levels, yet did not trigger classical apoptosis or significant oxidative stress (ROS production). Cytotoxicity assessment in CHO-K1 cells revealed an SI of 11.21, indicating a favorable safety margin for future optimizations. These findings establish (**59**) as a promising lead compound for the development of novel trypanocidal agents, reinforcing the potential of fluorinated benzothiazole derivatives in the rational design of drugs for Chagas disease.

Freitas and co-workers (2020) designed, synthesized, and evaluated sixteen novel 1,3,4-thiadiazole derivatives, belonging to the *N*-aminobenzyl and *N*-arylhydrazone series, to assess their trypanocidal activity against the trypomastigote form of *T. cruzi* [110]. Among the tested compounds, four exhibited promising activity, with compound (**60**) emerging as the most potent, displaying an IC_50_ of 3.6 µM and an SI of 26.9. Compound (**61**) also demonstrated a favorable balance between efficacy and selectivity, with an IC_50_ of 6.7 µM and SI of 66.1, while compound (**62**) showed an IC_50_ of 10.2 µM and SI of 66.6. Compound (**63**), although less potent, retained a considerable selectivity profile, exhibiting an IC_50_ of 16.6 µM and SI of 24.2 (Figure 7). Compounds (**61**) and (**62**) displayed the highest selectivity, establishing themselves as promising candidates for the development of new therapeutic agents against Chagas disease. Notably, compound (**63**) induced cellular effects comparable to those of posaconazole, a drug that disrupts the parasite’s sterol biosynthesis pathway. Further evidence supporting its potential mechanism of action was provided by electron microscopy analyses, which revealed morphological alterations in the kinetoplast and a reduction in flagellar length, suggesting structural damage to the parasite. Intracellular amastigote assays confirmed the ability of compounds (**63**) and (**62**) to significantly reduce the parasite burden without compromising the viability of host cells, indicating their selective action against *T. cruzi*. Furthermore, cytotoxicity assays demonstrated that (**62**) and (**63**) exhibited low toxicity toward human cardiomyocytes (lethal concentration 50%, LC_50_ > 400 µM), whereas (**61**) and (**60**) displayed a higher cytotoxic profile.

Silva et al. (2019) reported the synthesis of a series of 37 new thiosemicarbazones and semicarbazones containing the 1,2,3-1H-triazole-isatin scaffold and evaluated their in vitro antitrypanosomal activity against *T. cruzi* [111]. In addition to biological screening, molecular docking and electrochemical studies were conducted to investigate the potential molecular interactions and mechanisms of action of the most promising compounds. The derivatives (**64**) and (**65**) exhibited the best activities, with IC_50_ values of 24.1 µM (SI > 11.6) and 38.6 µM (SI = 11.8), respectively (Figure 7). Molecular docking studies indicated that both compounds interact favorably with the enzymes cruzain and phosphodiesterase C (TcrPDEC), validated targets in *T. cruzi*. Compound (**64**) showed docking energies of −5.18 kcal/mol (cruzain) and −8.31 kcal/mol (TcrPDEC), while (**65**) exhibited −5.40 kcal/mol (cruzain) and −7.47 kcal/mol (TcrPDEC), suggesting a stronger interaction of (**64**) with phosphodiesterase C. Electrochemical analyses demonstrated that both compounds exhibit distinct redox processes, which may be related to their mechanism of action. Compound (**64**) presented an oxidation potential (EpA) of +0.913 V and a reduction potential (EpC) of −0.611 V, whereas compound (**65**) exhibited an EpA of +1.11 V and an EpC of −0.850 V. These profiles suggest that the compounds may interact with the parasite’s redox systems, in addition to direct interactions with enzymatic targets, contributing to their trypanocidal activity.

### 1.2. Sleeping Sickness

HAT, commonly known as sleeping sickness, is an NTD caused by protozoan parasites of the *Trypanosoma* genus, transmitted by the tsetse fly (*Glossina* spp.) [112,113]. Human infection is primarily attributed to two subspecies: *Trypanosoma brucei gambiense* (*T. b. gambiense*) (92% of cases), responsible for a chronic, slow-progressing form endemic to West and Central Africa, and *T. b. rhodesiense* (8%), which causes a rapidly progressing and often fatal acute form, prevalent in East and Southeast Africa [114,115].

The current therapeutic arsenal includes suramin, melarsoprol, pentamidine, eflornithine, NECT (nifurtimox–eflornithine combination therapy), and fexinidazole [116]. Among these, fexinidazole, a nitroheterocyclic prodrug, represents a milestone in HAT treatment due to its oral bioavailability and ability to penetrate the blood–brain barrier (BBB), making it effective for both the hemolymphatic and meningoencephalitic stages of the disease [117,118]. However, its clinical application is limited by dose-dependent gastrointestinal toxicity and the potential for resistance mechanisms associated with parasite nitroreductase activity [119].

Given these limitations, identifying novel heterocyclic scaffolds with improved pharmacological properties remains a priority. Thiazole, thiosemicarbazone, and semicarbazone derivatives have shown promise in targeting *T. brucei* metabolic pathways, including redox homeostasis and nucleic acid biosynthesis [120,121,122]. Rational drug design strategies seek to enhance selectivity and pharmacokinetics, paving the way for safer and more effective therapies for HAT.

**Figure 8:**
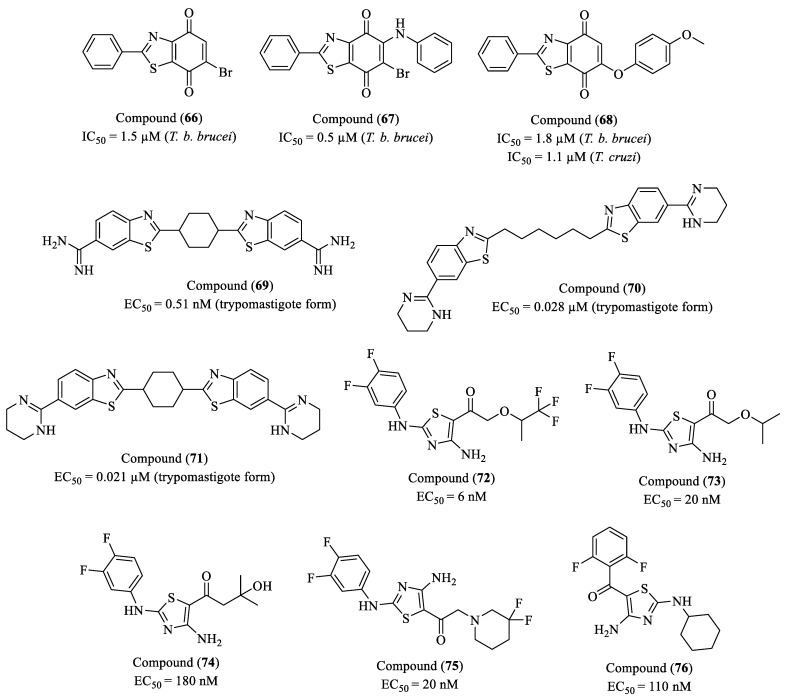
Structures of selected compounds (**66**–**76**) evaluated for their activity against *T. brucei*, the causative agent of human African trypanosomiasis. IC_50_ and EC_50_ values highlight structural features influencing potency and potential for further development.

Ballesteros-Casallas et al. (2023) synthesized four distinct series of p-quinones, including a third series containing thiazole derivatives, aiming to evaluate their activity against *T. brucei brucei*, *T. cruzi*, and *L. infantum* [123]. Among the thiazole-containing compounds, three exhibited excellent potency against *T. b. brucei*, namely compound (**66**) (IC_50_ = 1.5 μM, SI = 40), compound (**67**) (IC_50_ = 0.5 μM, SI < 22), and compound (**68**) (IC_50_ = 1.8 μM, SI = 43). Notably, compound (**68**) also demonstrated significant activity against *T. cruzi* (IC_50_ = 1.1 μM, SI = 71), emerging as one of the most selective derivatives in the series (Figure 8). However, none of the tested molecules exhibited meaningful activity against *L. infantum*, suggesting a greater specificity for the trypanosomatids *T. brucei* and *T. cruzi*. Structural analysis revealed that specific functional groups present in the thiazole derivatives directly influenced their biological efficacy. In the case of compound (**67**), the combination of the thiazole core with a bromine and an aniline moiety was associated with high potency against *T. b. brucei*. Meanwhile, compound (**68**), which displayed activity against *T. cruzi*, features a methoxy-phenyl substitution that appears to be critical for its selectivity. The incorporation of bulky substituents enhanced antiparasitic activity, while electronic modulation played a key role in both potency and selectivity. Additionally, mechanistic studies indicated that these *p*-quinones exert their activity by inducing oxidative stress in the parasites, leading to redox homeostasis disruption.

Racané and colleagues (2023) designed and synthesized a series of symmetric bis-6-amidino-benzothiazole derivatives containing aliphatic central linkers, which were tested for their antiparasitic activity against *T. brucei* [124]. The study focused on the relationship between structure, physicochemical properties, and in vitro efficacy, comparing the compounds to the reference drug fexinidazole. Compound (**69**) exhibited exceptional trypanocidal activity, with an EC_50_ in the sub-nanomolar range (0.00051 µM) and an SI exceeding 26,000, highlighting its outstanding potency and safety profile. Its structure, featuring unsubstituted amidine groups and a cyclohexane linker, was identified as crucial for its high efficacy. Additionally, (**69**) demonstrated nanomolar activity against asexual blood-stage forms of *P. falciparum*, suggesting potential cross-activity against protozoan parasites. Compound (**70**) also displayed notable trypanocidal activity, with an EC_50_ of 0.028 µM, a LogP of 4.23, and an SI greater than 4600, despite following a different SAR than the amidine-type molecules. Another promising derivative, (**71**), which differs from (**69**) in the spacing of its substituents, exhibited an EC_50_ of 0.021 µM, a LogP of 3.9, and an SI above 6200, making it a strong candidate for further structural modifications aimed at optimizing selectivity and activity (Figure 8). Only compound (**69**) was evaluated in in vivo assays, using a murine model of stage 1 *T. brucei* infection with bioluminescent luciferase expression. All mice treated with a single dose of 20 mg/kg were completely cured, with no evident signs of toxicity.

Cleghorn and co-workers (2023) reported the development and optimization of a series of diaminothiazole derivatives as potential therapeutic agents for HAT [125]. The study employed a SAR-driven approach, integrating phenotypic screening and mechanistic studies to elucidate the molecular targets involved. Initially designed as *Tb*GSK3 inhibitors, subsequent mechanistic investigations revealed that these compounds predominantly targeted inositol-tetrakisphosphate 1-kinase (ITPK1), a key enzyme in the inositol biosynthetic pathway, significantly altering their pharmacological profile. Following systematic SAR optimization, five lead compounds were selected based on potency, selectivity, metabolic stability, BBB penetration, and cytocidal versus cytostatic activity. Among them, compound (**72**) exhibited the highest potency, with an EC_50_ of 6 nM (0.006 μM), attributed to the introduction of a trifluoromethyl group, which enhanced target affinity without compromising selectivity or metabolic stability. Compound (**73**), with an EC_50_ of 20 nM (0.02 μM), demonstrated superior BBB permeability, likely due to its isobutoxy moiety, which conferred a 10-fold increase in activity compared to precursor analogs. Compound (**74**), displaying an EC_50_ of 180 nM (0.18 μM), was prioritized for in vivo evaluation due to its favorable BBB penetration properties. However, its predominantly cytostatic activity, rather than cytocidal, precluded further clinical progression. Compound (**75**), with an EC_50_ of 20 nM (0.02 μM), exhibited excellent potency and selectivity, attributed to strategic piperidine modifications, which optimized target engagement while mitigating mammalian cell toxicity. Lastly, compound (**76**), with an EC_50_ of 110 nM (0.11 μM), demonstrated in vivo efficacy, yet its rapid metabolic degradation rendered it unsuitable for advanced development (Figure 8).

**Figure 9:**
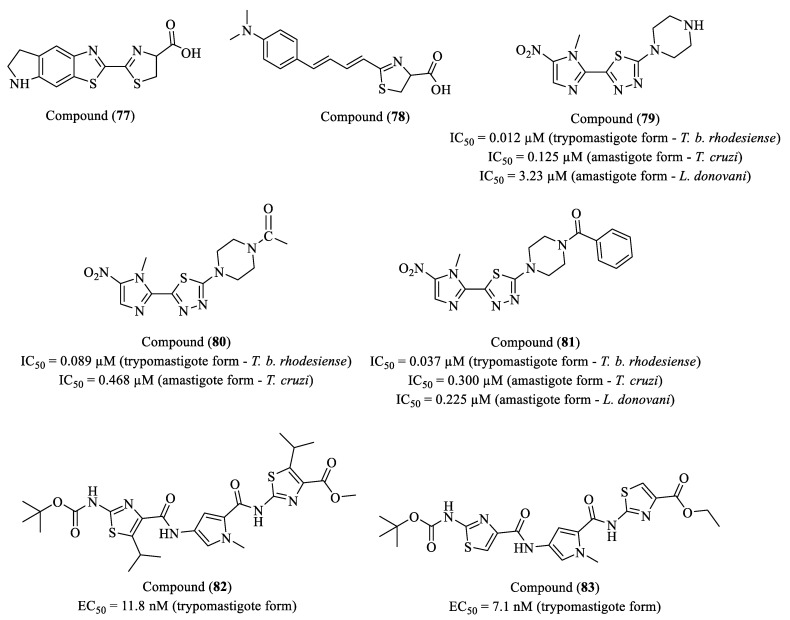
Chemical structures of selected compounds (**77**–**83**) evaluated for their activity against *T. brucei* and *T. cruzi*. IC_50_ and EC_50_ values highlight their potency against different parasite forms, with key structural features influencing target interaction, bioavailability, and selectivity. The presence of nitroaromatic, heterocyclic, and lipophilic moieties suggests diverse mechanisms of action relevant for the treatment of trypanosomiasis.

Hendrickx and co-workers (2022) investigated D-luciferin analogs, (**77**) and (**78**), as bioluminescent substrates for detecting parasitic infections in experimental and naturally transmitted models of visceral and cutaneous leishmaniasis and African trypanosomiasis (Figure 9) [126]. The study compared their in vitro and in vivo efficacy against D-luciferin, the gold standard substrate. Compound (**77**) contains a cyclic amino group, which enhances its cell and tissue permeability, while (**78**) features a benzothiazole substitution with an aromatic group, designed to increase luminogenic efficiency and tissue penetration. In vitro, (**77**) generated stronger bioluminescent signals than D-luciferin and (**78**) at higher concentrations, whereas (**78**) produced more intense signals at lower concentrations. However, in vivo studies revealed that (**78**) was unsuitable for parasitic infection monitoring due to high background signals in the liver, compromising the results’ interpretation. In contrast, (**78**) maintained high efficacy in vivo and was effective at doses up to 20 times lower than D-luciferin (7.5 mg/kg vs. 150 mg/kg). Compound (**77**) also crossed the BBB more efficiently, producing stronger bioluminescent signals in the brain, enabling more sensitive infection tracking. Although (**78**) exhibited a superior performance in vitro, its in vivo application was limited by excessive hepatic background signals. The study highlights that (**77**) is a highly effective and broadly applicable bioluminescent substrate for in vivo parasitic infection monitoring, reducing the required substrate dose and enhancing detection sensitivity.

Mousavi and colleagues (2022) synthesized new 2-(nitroaryl)-5-substituted-1,3,4-thiadiazole derivatives as potential antiparasitic agents. Among the most potent and selective compounds, (**79**), (**80**), and (**81**) exhibited high in vitro efficacy against bloodstream trypomastigotes of *T. b. rhodesiense* with low cytotoxicity in L6 cells and were compared to the reference drugs miltefosine and BZN (Figure 9). Compound (**79**) displayed the highest in vitro potency, with an IC_50_ of 0.012 µM and an SI of 11,703. Compound (**80**) exhibited an IC_50_ of 0.089 µM and an SI above 3000, while (**81**), from the same chemical class, presented an IC_50_ of 0.037 µM and an SI of 5167. These compounds were also evaluated against the amastigote forms of *T. cruzi* and *L. donovani*. Compound (**79**) showed IC_50_ values of 0.125 µM and 3.23 µM, respectively. Compound (**81**) exhibited IC_50_ values of 0.300 µM and 0.188 µM, while compound (**80**) displayed an IC_50_ of 0.468 µM against *T. cruzi*. Despite the remarkable in vitro potency of (**79**), in vivo assays using an acute HAT infection model revealed that (**81**) had the best overall performance. All infected mice were treated for four days with 50 mg/kg via intraperitoneal (IP) injection, and (**81**) demonstrated the highest cure rate and survival after 60 days. Compound (**79**) also showed a high cure rate; however, one of the treated mice died on day six and was excluded from the experimental group. Meanwhile, (**80**) achieved a 75% cure rate and also exhibited good survival after 60 days. The combination of high efficacy against multiple parasitic infections and low toxicity makes (**81**) a highly promising candidate for further development, with potential as a broad-spectrum antiparasitic treatment [127].

Franco et al. (2020) investigated distamycin analogs that selectively interact with AT-rich DNA regions, blocking the cell cycle of *T. brucei* by interfering with mitochondrial DNA replication [128]. To evaluate their therapeutic potential, the authors compared their efficacy with the reference drugs nifurtimox, suramin, and diminazene aceturate (DAC). Among the synthesized compounds, tri-heterocyclic derivatives (**82**) and (**83**) exhibited exceptional potency, with EC_50_ values below 20 nM and selectivity indices (SI) exceeding 5000 relative to murine macrophages (Figure 9). Notably, both compounds blocked mitochondrial DNA replication without compromising its integrity, representing a distinct mode of action compared to DAC, which induces mitochondrial DNA degradation. Cell cycle analysis indicated that the replication blockade is associated with the S phase, leading to cell cycle arrest and a trypanostatic effect. Additionally, comparative studies revealed that these compounds exhibit lower affinity for various DNA templates compared to DAC, suggesting a differentiated binding mechanism.

### 1.3. Leishmaniasis

Leishmaniasis is a neglected parasitic disease caused by protozoa of the *Leishmania* genus and transmitted by phlebotomine sandflies (*Phlebotominae*) [129,130,131]. The disease is endemic in 99 countries, affecting 12 million people and placing approximately 1 billion individuals at risk [132,133,134]. It manifests in three main clinical forms: cutaneous leishmaniasis (CL), mucocutaneous leishmaniasis (MCL), and visceral leishmaniasis (VL) [130,135,136]. CL is the most prevalent form, with 1 million new cases annually, while VL, caused by the *L. donovani* complex, has a 90% fatality rate if left untreated [137]. The most affected countries include India, Sudan, Brazil, and Kenya [134].

Current treatment options are hindered by high toxicity, prolonged administration regimens, and emerging parasite resistance, emphasizing the need for novel therapeutic strategies [138,139,140]. Molecular targets such as cysteine protease B (CPB), essential for parasite virulence, and trypanothione reductase (TR), involved in redox homeostasis, have been identified as promising avenues for drug development [141,142,143,144]. In this context, heterocyclic compounds with thiazole, thiosemicarbazone, and semicarbazone scaffolds have shown inhibitory potential against CPB and TR, disrupting essential parasite survival pathways [145,146].

**Figure 10:**
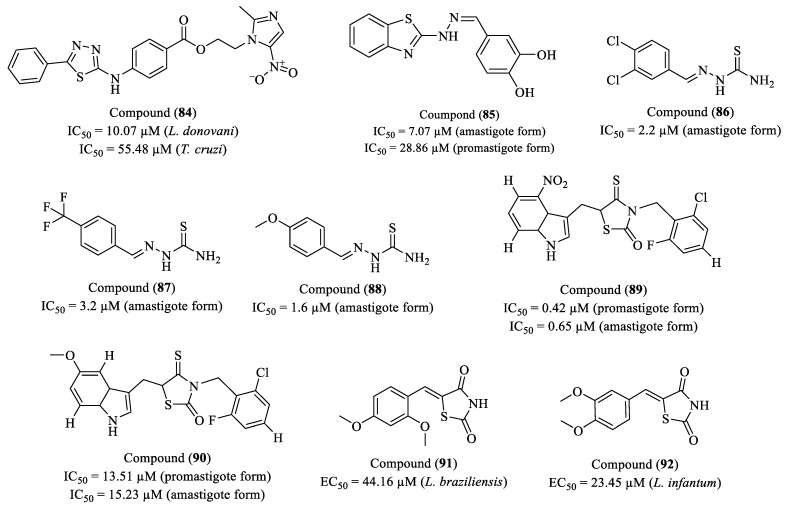
Selected thiazole, thiosemicarbazone, and semicarbazone derivatives (compounds **84**–**92**) evaluated for their antileishmanial activity. Heterocyclic cores, halogenated substituents, and aromatic moieties contribute to differential bioactivity across parasite forms.

Mijoba and co-workers (2024) synthesized six novel benzoate imidazo-1,3,4-thiadiazole derivatives, applying molecular repositioning and hybridization strategies, and assessed their antiparasitic activity against *L. donovani* and *T. cruzi* (Y strain) [147]. Among the tested compounds, compound (**84**) exhibited the most promising activity, achieving 71.42% inhibition of *L. donovani* and 58.35% inhibition of *T. cruzi*, with IC_50_ values of 10.07 µM and 55.48 µM, respectively (Figure 10). Furthermore, compound (**84**) demonstrated a high selectivity profile, with an SI of 49.65 for *L. donovani*, indicating notable antiparasitic potential alongside low cytotoxicity in Vero cells (CC_50_ > 500 µM). For comparison, the reference compounds presented the following results: BZN exhibited 45.39% inhibition against *T. cruzi*, with no IC_50_ reported, while amphotericin B (Aph) showed 62.44% inhibition against *L. donovani*, with an IC_50_ of 0.33 µM. These findings suggest that the strategic hybridization of structural elements from fexinidazole and metronidazole played a key role in enhancing antiparasitic activity, particularly when the thiadiazole core was modified with aromatic rings containing low-polarity substituents.

Coimbra et al. (2024) synthesized a series of benzothiazole derivatives and evaluated their in vitro activity against *L. amazonensis*, as well as their cytotoxicity in murine macrophages [148]. Compound (**85**) exhibited the best activity, with an IC_50_ of 7.7 μM against amastigotes and 28.86 μM against promastigotes, surpassing miltefosine (IC_50_ = 12.52 μM) (Figure 10). Mechanistic studies indicated that the compound reduced mitochondrial membrane potential by up to 77%, promoted the accumulation of neutral lipids, and did not induce ROS production. Moreover, it did not alter the integrity of the parasite plasma membrane. SAR analysis revealed that the presence of hydroxyl groups at positions 3 and 4 of the aromatic ring was essential for activity, likely due to hydrogen bonding interactions with the biological target. Substitution with a methoxy group at position 4 improved activity, whereas multiple methoxy substitutions reduced efficacy, suggesting an unfavorable steric effect. In silico absorption, distribution, metabolism, excretion, and toxicity (ADMET) analysis indicated good intestinal absorption, moderate permeability across the BBB, and low predicted toxicity, along with an absence of carcinogenic potential. The compound did not significantly inhibit cytochrome P450 enzymes, suggesting a favorable metabolic profile. The results demonstrate that compound (**85**) is a strong candidate for preclinical studies as an antileishmanial agent, with a promising mechanism of action and a pharmacokinetic profile suitable for further investigation.

Aquino and researchers (2022) designed, synthesized, and evaluated the biological activity of thiosemicarbazones against *L. chagasi* amastigotes (MCAN/BR/89/BA262), in addition to investigating the SAR of these compounds and exploring potential mechanisms of action through molecular docking to predict interactions with the TR enzyme [149]. Among the compounds tested, derivatives (**86**), (**87**), and (**88**) exhibited significant activity against the amastigote form of the parasite, along with low cytotoxicity toward J774.A1 macrophages (Figure 10). Compound (**88**) displayed an IC_50_ of 1.6 µM and an SI of 44, making it the most promising candidate in the series. Derivative (**86**) exhibited an IC_50_ of 2.2 µM and an SI of 31, whereas compound (**87**) showed an IC_50_ of 3.2 µM and an SI of 25. The SAR analysis highlighted that the presence of para-positioned substituents on the aromatic ring enhances the biological activity of the thiosemicarbazones. Molecular docking studies indicated that the most active compounds bind within the active site of the TryR enzyme, engaging in key interactions with Ser14, Tyr198, Met333, and Ala338. Notably, compound (**88**), in addition to exhibiting the highest in vitro activity, demonstrated an additional hydrogen bond interaction with Tyr198, which may account for its enhanced potency.

Gouveia and colleagues (2022) investigated the cytotoxicity and antiparasitic potential of two series of thiazolidine derivatives against *L. infantum*, identifying compound (**89**) as the most promising [150]. This analogue exhibited an IC_50_ of 0.42 μM against promastigotes and 0.65 μM against amastigotes, with an SI of 13.11, indicating a favorable therapeutic window. Ultrastructural analysis revealed significant morphological alterations, including flagellar shortening, cytoplasmic vacuolization, and mitochondrial swelling, suggesting a possible mechanism of action associated with mitochondrial dysfunction. Additionally, the induction of NO production in macrophages may contribute to its leishmanicidal effect, reinforcing its immunomodulatory potential. Compound (**90**), containing a methoxy (-OCH_3_) group, also demonstrated moderate activity (IC_50_ = 13.51 μM and 15.23 μM), though with lower selectivity. SAR analysis indicated that the presence of the nitro (-NO_2_) group in (**89**) was critical for its high efficacy, possibly acting as an intracellular bioactivator. The incorporation of halogens, such as fluorine and chlorine, may have influenced both permeability and molecular interactions with the biological target. These findings position compound (**89**) as a promising candidate for further development of antileishmanial therapies, combining potent antiparasitic activity, cellular selectivity, and favorable pharmacokinetic properties (Figure 10).

Neri and co-workers (2020) evaluated the antileishmanial activity of thiazolidine-2,4-dione derivatives against *L. infantum* and *L. braziliensis*, investigating their interaction with pteridine reductase 1 (PTR1), a validated therapeutic target [151]. Among the synthesized compounds, (**91**) exhibited the highest potency against *L. infantum* (EC_50_ = 23.45 μM, SI = 1.85), while (**92**) showed superior efficacy against *L. braziliensis* (EC_50_ = 44.16 μM, SI = 0.76) (Figure 10). However, both compounds displayed low selectivity, exhibiting cytotoxicity toward human cells, which limits their pharmacological potential. Kinetic characterization revealed that (**91**) has a higher affinity for LmPTR1 (Kd = 23.12 μM), which may explain its enhanced potency against *L. infantum*. In contrast, compound (**92**), with a higher dissociation constant (Kd = 43.42 μM), exhibited weaker interaction with PTR1 yet retained efficacy against *L. braziliensis*. It is suggested that lipophilic substituents at the meta and/or para positions of the benzylidene ring enhance this interaction, thereby increasing antileishmanial activity.

**Figure 11:**
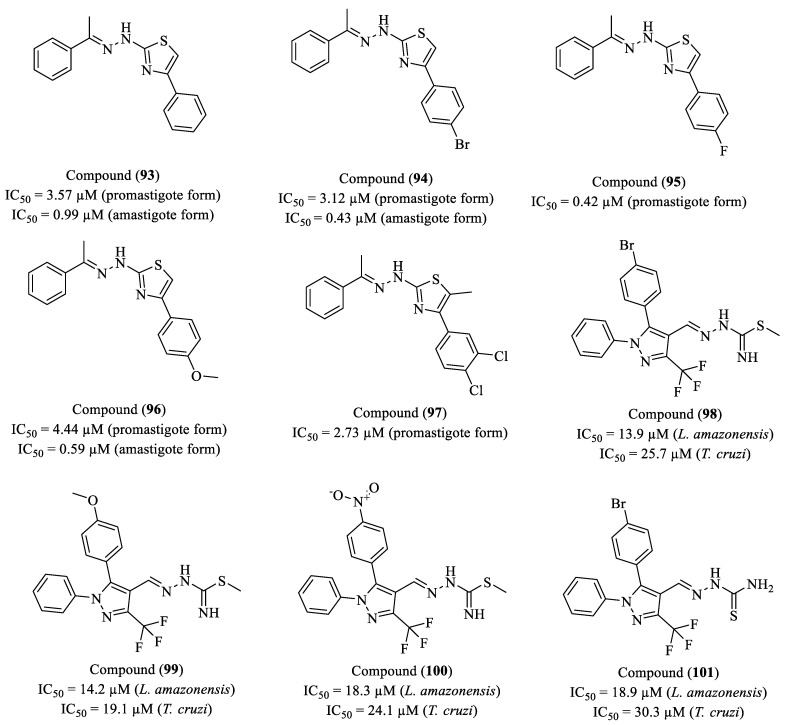
A diverse set of thiazole, thiosemicarbazone, and semicarbazone derivatives (compounds **93**–**101**) evaluated for their antileishmanial activity. IC_50_ values against promastigote and amastigote forms of *Leishmania* spp. reveal the influence of halogenation, electron-donating groups, and aromatic substitutions on bioactivity.

De Oliveira et al. (2020) investigated the leishmanicidal activity of thiazole-containing compounds against *L. infantum* [152]. Ten compounds were evaluated, including five thiazopyridine derivatives and five thiazolacetylpyridine derivatives, for their cytotoxicity in peritoneal macrophages from BALB/*c* mice and their efficacy against the promastigote and amastigote forms of the parasite. Among the tested compounds, (**93**), (**95**), and (**97**) exhibited the highest potency against promastigotes, with IC_50_ values of 3.57 µM, 0.42 µM, and 2.73 µM, respectively. Against amastigotes, compounds (**93**), (**94**), and (**96**) demonstrated the best efficacy, with IC_50_ values of 0.99 µM, 0.43 µM, and 0.59 µM, respectively (Figure 11). Compound (**94**) exhibited the best selectivity indices (SI_pro_ = 18.94; SI_ama_ = 137.37), reducing nearly 100% of macrophage infection at 1 mg/mL. Additionally, ultrastructural analysis revealed mitochondrial alterations, nuclear displacement, and membrane damage, suggesting an apoptosis-like cell death mechanism. These results indicate that the thiazolacetylpyridine series compounds, particularly (**95**), are promising candidates for the development of new therapies against visceral leishmaniasis.

Camargo and researchers (2020) synthesized a series of trifluoromethylated compounds and evaluated their activity against the promastigote forms of *L. amazonensis* and epimastigote forms of *T. cruzi* [153]. Compounds (**98**), (**99**), (**100**), and (**101**) exhibited the best antiparasitic activity profiles and selectivity (Figure 11). Compound (**98**), containing a bromine group in the para position of the aromatic ring, showed the highest potency among the *S*-methyl thiosemicarbazones, with an IC_50_ of 13.9 µM for *L. amazonensis* and 25.7 µM for *T. cruzi*, along with an SI of 10.55 for *L. amazonensis* and 5.71 for *T. cruzi*. Compound (**99**), containing a methoxy group, exhibited an IC_50_ of 14.2 µM for *L. amazonensis* and 19.1 µM for *T. cruzi*, with SI values of 8.65 for *L. amazonensis* and 6.43 for *T. cruzi*. The derivative (**101**), bearing a nitro group, displayed an IC_50_ of 18.3 µM for *L. amazonensis* and 24.1 µM for *T. cruzi*, with SI values of 12.10 for *L. amazonensis* and 9.19 for *T. cruzi*. Compound (**101**), belonging to the thiosemicarbazone series and also containing a bromine group, presented an IC_50_ of 18.9 µM for *L. amazonensis* and 30.3 µM for *T. cruzi*, with SI values of 8.98 for *L. amazonensis* and 5.60 for *T. cruzi*. The SAR analysis revealed that the presence of bulky substituents in the para position of the aromatic ring positively influenced biological activity, with bromine-, methoxy-, and nitro-containing derivatives being the most effective. The conversion of thiosemicarbazones into their *S*-methylated form resulted in increased compound potency, making them approximately 1.5 times more active than their non-methylated counterparts, highlighting the influence of this group on molecular interactions with biological targets. Additionally, compounds containing nitro and methoxy groups exhibited greater selectivity, suggesting reduced toxicity toward host cells.

### 1.4. Malaria

Malaria is a parasitic disease transmitted by female Anopheles mosquitoes infected with protozoa of the *Plasmodium* genus (*P. falciparum*, *P. vivax*, *P. malariae*, *P. ovale*, and *P. knowlesi*) [154]. The disease remains endemic in 83 countries, with an estimated 260 million cases in 2023 [155,156], including transmissions via mosquito bites, blood transfusions, and accidental exposure to contaminated needles [157,158,159]. During the same period, 597,000 deaths were reported, with 94% of cases (246 million) and 95% of fatalities (569,000) occurring in Africa, where 76% of deaths affect children under five years old, primarily in Nigeria, the Democratic Republic of the Congo, Niger, and Tanzania [160].

The current standard treatment relies on artemisinin-based combination therapies (ACTs), along with other antimalarial agents such as primaquine and chloroquine [161,162,163]. However, the emergence of drug-resistant *P. falciparum* strains has significantly compromised treatment efficacy, underscoring the urgent need for novel therapeutic strategies [164,165,166]. In this context, heterocyclic scaffolds, including thiazole, thiosemicarbazone, and semicarbazone derivatives, have emerged as promising alternatives for the development of next-generation antimalarial agents [167,168]. These structures offer high synthetic versatility and can target critical *Plasmodium* enzymes, such as dihydrofolate reductase (DHFR) in folate metabolism, hemozoin formation in heme detoxification, nucleotide biosynthesis, and ion transport mechanisms [169]. Modulation of these targets can effectively disrupt parasite replication, positioning these compounds as attractive candidates for the development of more potent and selective antimalarial drugs.

**Figure 12:**
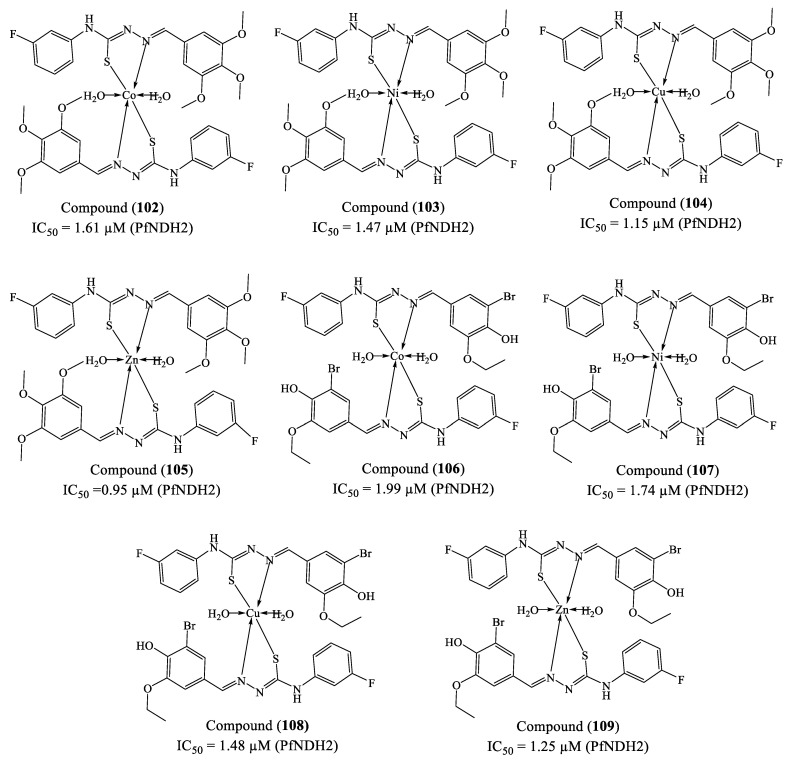
Metal-based complexes (compounds **102**–**109**) evaluated for their antiplasmodial activity against *P. falciparum*. The IC_50_ values against the *Pf*NDH2 strain highlight the role of metal coordination, ligand environment, and hydration state in modulating bioactivity.

Kumar et al. (2024) investigated the synthesis and biological evaluation of new transition metal complexes containing thiosemicarbazone ligands [170]. The complexes (**102**), (**103**), (**104**), (**105**), (**106**), (**107**), (**108**), and (**109**) exhibited significant in vitro antimalarial activity against *P. falciparum* (3D7 strain), inhibiting schizont maturation with IC_50_ values ranging from 0.95 to 1.99 µM, comparable to quinine (IC_50_ = 0.26 µM) (Figure 12). Among them, the Zn(II) complex (**105**) displayed the highest potency, with an IC_50_ close to the reference drug quinine (IC_50_ = 0.26 µM). Molecular docking studies were conducted against *Pf*NDH2 (PDB ID: 5JWA), a key target for new antimalarial agents. The binding energies ranged from −188.171 kcal/mol (**103**) to −199.749 kcal/mol (**105**) −197.682 kcal/mol, indicating high affinity. The complexes formed stable interactions with critical protein residues, including Asn92, Ser90, Gly87, Leu89, and Lys523, suggesting strong potential for enzyme inhibition. The ADMET analysis indicated good intestinal absorption, low hepatotoxicity, high membrane permeability, and no mutagenicity, suggesting favorable pharmacokinetics and safety. Thus, these transition metal complexes appear to be potential candidates for the development of novel antimalarial agents, with Zn(II) complexes (**105**) and (**109**) standing out as the most effective and least toxic. However, further in vivo studies are necessary to confirm its clinical applicability.

**Figure 13:**
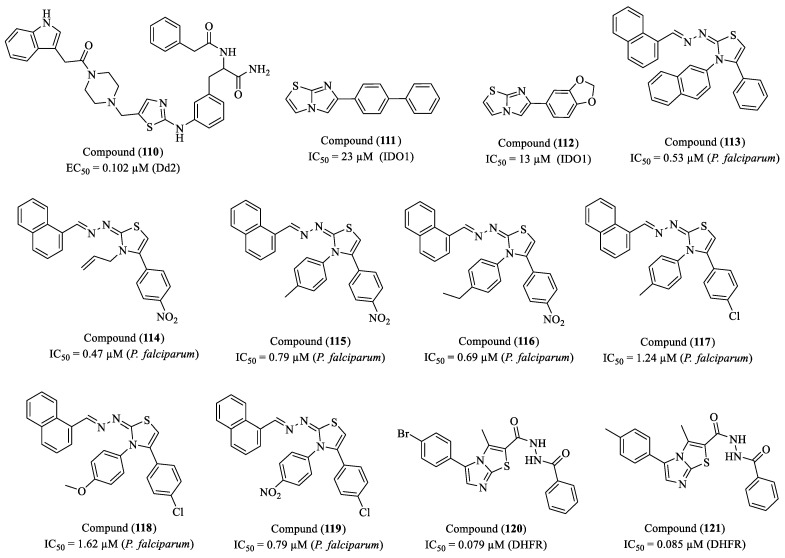
Selected antiplasmodial compounds (**110**–**121**) evaluated for their activity against *P. falciparum* and drug-resistant strains. The IC_50_ and EC_50_ values highlight key structural features, including heterocyclic scaffolds, nitroaromatic and halogenated substituents, which influence potency and selectivity.

Rayala et al. (2023) investigated the synthesis and antiplasmodial activity of compounds containing thiazole and piperazine rings, two heterocyclic structures widely used in medicinal chemistry [171]. The authors synthesized a library of piperazine–thiazole compounds using parallel and solid-phase synthesis methods. Screening against *P. falciparum* (chloroquine-resistant Dd2 strain) revealed compounds with promising antiplasmodial activity. The most effective compound (**110**) exhibited an EC_50_ of 102 nM (0.102 µM) and an SI greater than 140, suggesting significant therapeutic potential. The compound’s high selectivity indicates low toxicity to human cells, as tested on the HepG2 cell line (Figure 13). This suggests a favorable safety profile, a crucial factor in the development of new antimalarial drugs.

Singh and co-researchers (2023) evaluated the inhibitory activity of IDO1 (indoleamine 2,3-dioxygenase 1), an immunomodulatory enzyme that catalyzes the first and rate-limiting step of the kynurenine pathway in *L*-tryptophan metabolism [172]. IDO1 overexpression has been associated with the progression of several diseases, including cancer, malaria, and multiple sclerosis. In this study, compounds (**111**) and (**112**) exhibited IC_50_ values of 23 µM and 13 µM, respectively, demonstrating potential as IDO1 inhibitors (Figure 13). Additionally, both compounds were found to be non-cytotoxic to HEK293 cells at concentrations of up to 100 µM, as assessed by the Alamar Blue cell viability assay. Molecular docking studies were conducted using five crystal structures of IDO1 (PDB IDs: 6R63, 5XE1, 4PK6, 6E40, and 5EK2). The analysis revealed that several compounds achieved reasonable docking scores in at least two of these structures, suggesting that they adopt a similar orientation to the co-crystallized ligands. The proposed structural model indicates that effective IDO1 inhibitors should feature a heterocyclic core capable of interacting with the Fe(II) ion of the heme group, as well as aromatic substitutions that facilitate interactions with hydrophobic residues in the active site. These findings suggest that imidazo [2,1-b]thiazole-based compounds hold promise as novel IDO1 inhibitors and could serve as a foundation for further structural optimizations aimed at developing more potent therapeutic candidates.

The study conducted by Santos et al. (2023) explores the synthesis, characterization, and biological activities of new naphthyl-thiazole derivatives, with an emphasis on thiosemicarbazones and thiazoles [173]. Thirteen thiosemicarbazones and sixteen thiazoles were synthesized, and their properties were evaluated regarding antioxidant and antiparasitic activities. The study revealed that while the thiosemicarbazones did not show efficacy in inhibiting the growth of *P. falciparum*, the thiazoles exhibited promising activity. Specifically, four compounds—(**113**), (**114**), (**115**), and (**116**)—showed IC_50_ values comparable to the standard drug chloroquine (IC_50_ = 0.075 μM) (Figure 13). Compound (**113**) presented an IC_50_ of 0.53 μM, while compounds (**114**), (**115**), and (**116**) exhibited IC_50_ values of 0.47 μM, 0.79 μM, and 0.69 μM, respectively. The presence of the 4-nitrophenyl group in compounds (**114**), (**115**), and (**116**) is a relevant factor, given that the nitro group is widely recognized for its significant contribution to molecules with antiparasitic profiles, justifying its choice as a substituent group. The compounds underwent in silico analyses of ADMET properties, yielding results that indicate good intestinal absorption (>70%), making them promising candidates for oral administration. Compounds (**113**) and (**116**) showed a greater tendency for distribution in plasma than in tissues.

Silva and collaborators (2023) investigated the antimalarial potential of 4-(4-chlorophenyl)thiazole compounds through in silico ADMET property predictions and in vitro assays against the chloroquine-sensitive *P. falciparum* 3D7 strain [174]. The compounds exhibited low toxicity in mouse splenocytes, promoting cell activation by increasing NO production without inducing cell death. Regarding antimalarial activity, compounds (**117**) (IC_50_ = 1.24 μM), (**118**) (IC_50_ = 1.62 μM), and (**119**) (IC_50_ = 0.79 μM) stood out, with the latter being the closest to chloroquine (IC_50_ = 0.75 μM) (Figure 13). These results indicate that the compounds possess favorable pharmacokinetic properties, low toxicity in mammalian cells, and selectivity against the parasite, making them promising candidates for the development of new antimalarial drugs. However, additional studies, including in vivo assays, are necessary to confirm their efficacy and safety.

Ewida and co-workers (2021) developed and evaluated a new class of 3-methyl-imidazo[2,1-b]thiazole derivatives as inhibitors of DHFR, an enzyme essential for purine and thymidylate synthesis, making it a key target in the treatment of bacterial infections, malaria, and various types of cancer [175]. A series of analogs were synthesized and characterized, among which compounds (**120**) and (**121**) exhibited potent inhibitory activity against DHFR, with IC_50_ values of 0.079 and 0.085 µM, respectively (Figure 13). These results are comparable to methotrexate (MTX, IC_50_ = 0.087 µM), a classic inhibitor of this enzyme widely used in cancer treatment. Molecular modeling studies indicated that compounds (**120**) and (**121**) have a high affinity for the DHFR active site, interacting with Arg22 and Phe31 residues through hydrogen bonding and π-π interactions, reinforcing the proposed mechanism of action. Computational analysis of ADMET parameters revealed that these compounds exhibit good intestinal absorption, high BBB penetration, and no hepatotoxicity potential. Furthermore, both compounds do not inhibit the CYP2D6 enzyme, minimizing the risk of adverse drug interactions.

**Figure 14:**
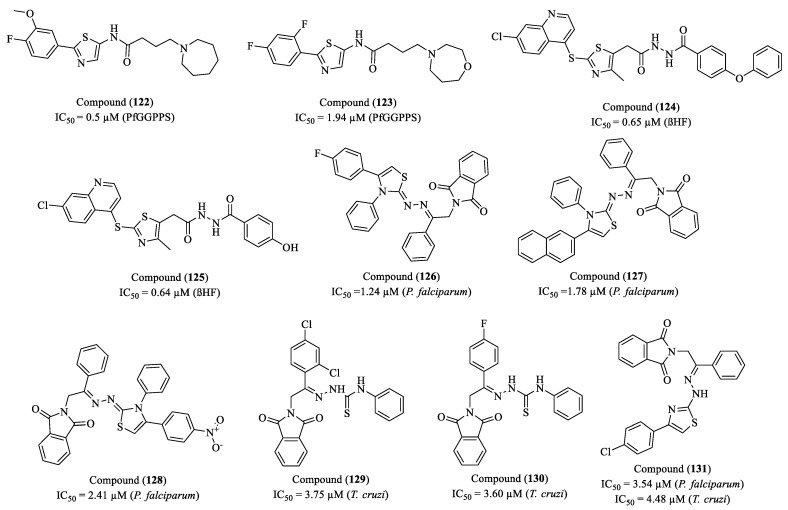
Diverse antiplasmodial compounds (**122**–**131**) evaluated against *P. falciparum* and related targets. IC_50_ values indicate the influence of structural features such as heterocyclic cores, halogenation, and functionalized side chains on bioactivity and selectivity.

The study by Kabeche and collaborators (2021) presents the synthesis and evaluation of a new series of thiazole-containing amides as selective non-bisphosphonate inhibitors of *P. falciparum* farnesyl/geranylgeranyl diphosphate synthase (*Pf*FPPS/GGPPS), an enzyme crucial for the parasite’s survival [176]. This enzyme is a promising target for antimalarial drug development due to its essential role in the isoprenoid biosynthesis pathway, which differs significantly from the mevalonate pathway in humans. Through SAR studies, the researchers developed derivatives of MMV019313, the first highly selective non-bisphosphonate inhibitor of *Pf*FPPS/GGPPS. Several compounds exhibited favorable in vitro absorption, distribution, metabolism, and excretion (ADME) profiles, with (**122**) (IC_50_ = 0.50 µM) standing out for its high inhibitory potency and lipophilic efficiency. The most promising compounds were subjected to pharmacokinetic studies in mice, with (**123**) demonstrating particularly favorable properties, including good systemic exposure and metabolic stability (Figure 14). Although (**123**) showed a slight reduction in inhibitory potency (IC_50_ = 1.94 µM) compared to other compounds in the series, its excellent lipophilicity and metabolic profile justify its selection as a lead candidate for further in vivo investigations.

Ramírez et al. (2021) synthesized and evaluated new 7-chloroquinoline-4-thiazoleacetic derivatives containing aryl-hydrazides with a focus on antimalarial activity [177]. The synthesis was carried out through molecular hybridization, coupling 2-[2-(7-chloroquinolin-4-ylthio)-4-methylthiazol-5-yl]acetic acid with various benzoyl hydrazines via Steglich esterification. The compounds were tested for their ability to inhibit β-hematin formation, a crucial process for *Plasmodium* survival. Derivatives (**124**) and (**125**) stood out as the most active, exhibiting IC_50_ values of 0.65 µM and 0.64 µM, respectively, comparable to chloroquine, which showed an IC_50_ of 0.18 µM (Figure 14). In in vivo assays, compounds (**124**) and (**125**) were tested in mice infected with *P. berghei* and demonstrated the ability to significantly reduce parasitemia and prolong animal survival. Compound (**124**) increased the average survival time to 21.4 days, while compound (**125**) extended it to 20.9 days. However, these values remain lower than those observed with chloroquine, which prolonged survival to 30 days and reduced parasitemia to 1.40%. Additionally, the compounds were evaluated for safety in human red blood cells, showing low hemolysis at concentrations of up to 1 mM, a safer profile than chloroquine, which induced 15.4% hemolysis at the same concentration.

Gomes and co-workers (2020) synthesized and evaluated the biological activity of a series of phthalimide–thiosemicarbazone and phthalimide–thiazole derivatives against *P. falciparum* (strain 3D7-GFP) and *T. cruzi* (strain Y) [178]. Additionally, they investigated potential mechanisms of action and analyzed the physicochemical properties and ADME profile in silico to assess their drug-like characteristics. Among the most active compounds, (**126**), (**127**), and (**128**) exhibited the highest potency against *P. falciparum*, with IC_50_ values of 1.24 µM (SI > 302.42), 1.78 µM (SI > 198.99), and 2.41 µM (SI = 79.79), respectively (Figure 14). Against *T. cruzi*, compounds (**129**) and (**130**) displayed the best activity, with IC_50_ values of 3.75 µM (SI = 37.46) and 3.60 µM (SI = 68.21), respectively. Moreover, (**131**) stood out for its dual antiparasitic activity, presenting an IC_50_ of 3.54 µM (SI = 50.28) against *P. falciparum* and an IC_50_ of 4.48 µM (SI = 39.73) against *T. cruzi*. This derivative demonstrated a promising broad-spectrum antiparasitic profile. Mechanistic studies revealed that (**126**) induced apoptosis and necrosis in *T. cruzi* trypomastigotes, as evidenced by flow cytometry analysis. Furthermore, SEM imaging showed that (**129**), (**130**), and (**131**) caused significant morphological alterations in the parasites, including cell deformation, flagellum shortening, and the presence of filamentous structures on the surface, along with cytoplasmic content leakage in *T. cruzi* treated with (**131**), an effect similar to that observed with BZN. Regarding ADME properties, the analyzed compounds complied with Lipinski’s rule of five, indicating favorable oral absorption and permeability. In silico predictions further suggested high gastrointestinal absorption for most compounds, whereas (**126**) displayed borderline values, indicating that its absorption might be variable. Additionally, none of the compounds exhibited significant CYP450 enzyme inhibition potential, minimizing the risk of drug–drug interactions.

**Figure 15:**
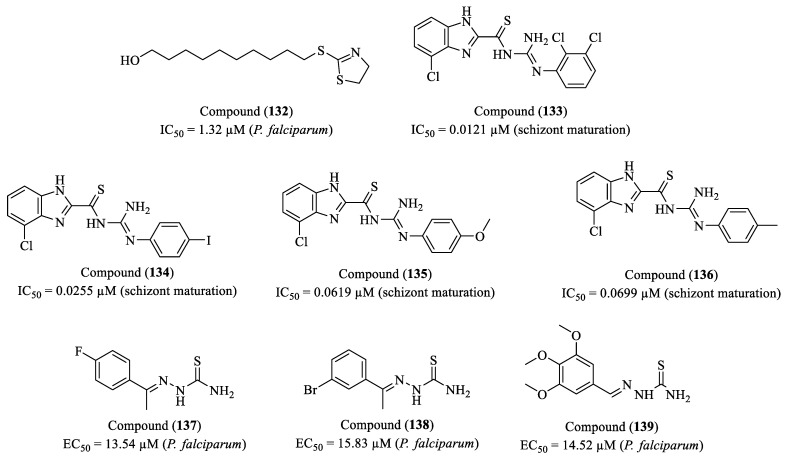
Antiplasmodial compounds (**132**–**139**) targeting *P. falciparum* and schizont maturation. IC_50_ and EC_50_ values highlight the impact of molecular features, including sulfur-containing moieties, halogenated heterocycles, and alkylated side chains, on bioactivity and selectivity.

The study by Silva et al. (2020) investigated the incorporation of (**132**), a synthetic analog of 3-alkylpyridine marine alkaloids, into an oil-in-water nanoemulsion for malaria treatment (Figure 15) [179]. The formulation was optimized using a 2^3^ factorial design and characterized in terms of stability, encapsulation efficiency, and release profiles at different pH levels. The nanoemulsion demonstrated high in vitro efficacy against *P. falciparum* (IC_50_ = 1.32 μM) and reduced in vivo parasitemia for 8 days without extending animal survival, possibly due to the compound’s short half-life. Despite its potential, pharmacokinetic adjustments are needed to improve the clinical efficacy of the nanoemulsion. It is suggested to enhance text cohesion and explore strategies to optimize the bioavailability of (**132**). Further studies and clinical trials will be essential to establish this approach as a promising alternative for malaria treatment.

Divatia and colleagues (2019) investigated the synthesis and antimalarial activity of novel thiosemicarbazone derivatives containing a benzimidazole moiety [180]. The study assessed the compounds’ ability to inhibit schizont maturation of *P. falciparum* in cultured human erythrocytes using an in vitro assay to determine IC_50_ values. The IC_50_ values were compared to reference drugs, including chloroquine (IC_50_ = 0.020 µg/mL) and quinine (IC_50_ = 0.268 µg/mL), allowing for an evaluation of the relative efficacy of the synthesized compounds. Subsequently, Nkungli et al. (2024) expanded this research by exploring the inhibitory potential of these compounds against falcipain-2 (FP2), a key enzyme in the parasite’s metabolism [181]. The study employed molecular docking, molecular dynamics simulations, and free energy calculations to analyze the interactions between the compounds and FP2, as well as to investigate potential covalent inhibition mechanisms. Compounds (**133**), (**134**), (**135**), and (**136**) exhibited extremely low inhibition constant (Ki) values, ranging from 5.94 × 10^−14^ to 2.59 × 10^−4^ nM, indicating a high binding affinity to FP2 (Figure 15). Additionally, binding free energy calculations (MM/GBSA) ranged from −30.32 to −17.17 kcal/mol, suggesting highly efficient enzyme–ligand interactions. Molecular dynamics simulations revealed that the interaction between (**133**) and FP2 remained stable, without causing significant structural changes to the enzyme. Furthermore, ONIOM calculations suggested that (**133**), and potentially (**134**) and (**135**), could act as covalent inhibitors of FP2, exhibiting viable reaction energy barriers in aqueous and organic environments. These findings provide valuable insights for the development of novel antimalarial drugs, highlighting BZD–thiosemicarbazone hybrid molecules as promising candidates for *P. falciparum* inhibition.

Matsa and researchers (2019) designed and evaluated a series of novel thiosemicarbazone derivatives against *P. falciparum* (3D7 strain) [182]. Among the synthesized compounds, three exhibited promising antimalarial activity: compound (**137**) (EC_50_ = 13.54 µM), compound (**138**) (EC_50_ = 15.83 µM), and compound (**139**) (EC_50_ = 14.52 µM) (Figure 15). The SAR analysis indicates that the incorporation of halogens and methoxy groups enhances antimalarial potency. The 4-fluorophenyl moiety in compound (**138**) and the 3-bromophenyl substituent in compound (**138**) suggest that halogenation can modulate key interactions with the biological target. Meanwhile, compound (**139**), featuring a 3,4,5-trimethoxybenzylidene system, stands out for its potential influence on lipophilicity and molecular recognition. Furthermore, meta-substitution, as observed in compound (**138**), appears to confer greater antimalarial activity compared to other positional isomers.

## 2. Perspectives

**Figure 16:**
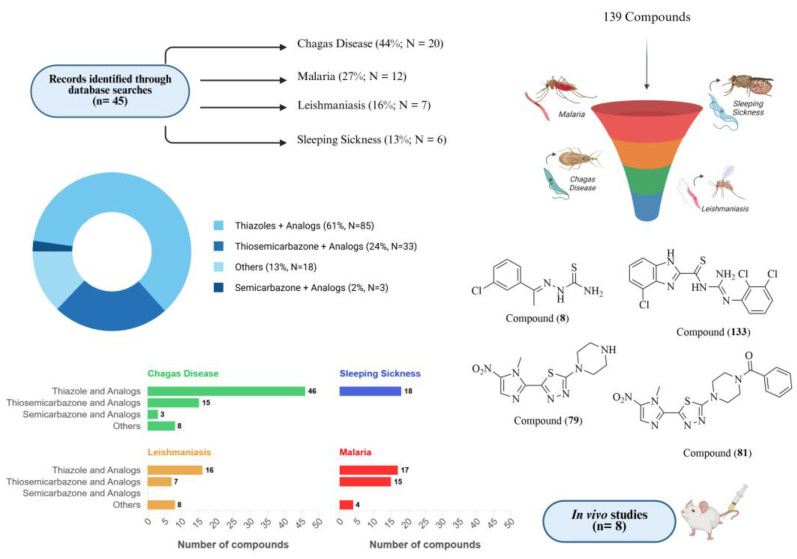
Literature-based screening, structural classification, and scaffold analysis of antiparasitic compounds for Chagas disease, malaria, leishmaniasis, and sleeping sickness.

This study provides a comprehensive analysis of antiparasitic compounds reported in the scientific literature for the treatment of NTDs, including Chagas disease, malaria, leishmaniasis, and sleeping sickness. A search in specialized databases identified 45 relevant studies, of which 44% (N = 20) focused on Chagas disease, 27% (N = 12) on malaria, 16% (N = 7) on leishmaniasis, and 13% (N = 6) on sleeping sickness. In total, 139 compounds were evaluated for their antiparasitic activity (Figure 16).

Structural classification revealed a predominance of thiazoles and their analogs, which accounted for 61% (N = 85) of the total. Thiosemicarbazones and their analogs represented 24% (N = 33), while 18 compounds were grouped under the “other” category, which included different structural classes, such as thiazolidinones (13%). Only three compounds (2%) belonged to the semicarbazone class and its analogs. The distribution of scaffolds across diseases reinforced the relevance of thiazoles. In Chagas disease, 46 thiazole-based compounds were identified, along with 15 thiosemicarbazones, 3 semicarbazones, and 8 compounds from other classes. For sleeping sickness, all 18 analyzed compounds were thiazole derivatives. In leishmaniasis, 16 compounds were classified as thiazoles, followed by 7 thiosemicarbazones and 8 compounds from another category. In malaria, 17 thiazoles were identified, along with 15 thiosemicarbazones and 4 compounds classified as “others”. These findings highlight thiazoles as a key scaffold in the development of therapeutic agents for NTDs.

Despite their therapeutic potential, the transition to in vivo studies remains limited, with only eight primary studies reporting in vivo evaluation of selected compounds. This gap between identifying promising candidates and advancing them through the pharmaceutical development pipeline highlights the scarcity of preclinical investigations. To address this, we expanded the discussion to include eleven thiazole derivatives assessed in vivo against *Trypanosoma* and *Plasmodium* species. These include compounds targeting bloodstream trypomastigotes (*T. brucei*, *T. cruzi*), blood-stage *Plasmodium*, and intracellular amastigotes. For instance, compound (**69**) achieved a complete cure in a murine model of *T. brucei* infection with a single 20 mg/kg dose, outperforming fexinidazole. Compounds (**79**–**81**) demonstrated high efficacy against bloodstream forms of *T. b. rhodesiense*, with compound (**81**) providing the highest survival rate. Compound (**30**) reduced parasitemia by approximately 80% in *T. cruzi*-infected mice at 1 mg/kg, while compound (**58**) exhibited moderate efficacy but also central nervous system effects. Antimalarial thiazoles (**124** and **125**) led to significant reductions in parasitemia and extended survival, with lower hemolysis compared to chloroquine. Compound (**132**), although effective in reducing *P. falciparum* parasitemia, did not extend survival, possibly due to a short half-life. It is also important to note that the compounds reported by Hendrickx et al. (2022) [126] were evaluated for in vivo imaging applications rather than therapeutic purposes and thus were not included in the mechanistic discussions.

To contextualize these findings, we performed a comparative assessment between the most promising thiazole-based compounds and reference drugs currently used for NTDs. Several candidates showed superior or comparable in vitro activity to benznidazole, miltefosine, or chloroquine, with improved selectivity indexes and reduced toxicity. For example, compounds (**79**) and (**81**) exhibited selectivity indexes greater than 5000 and 11,000, respectively, suggesting a favorable safety profile. These comparative results reinforce the potential of these compounds to overcome limitations associated with current treatments, such as toxicity, limited efficacy, and long treatment durations.

Nevertheless, there remains a significant gap between early-stage discovery and clinical application. A search on ClinicalTrials.gov identified only 15 studies involving thiosemicarbazones, 5 involving thiazoles, and 2 involving thiazolidinones—none of which targeted neglected tropical diseases [183]. Most of these studies were focused on cancer, highlighting the disparity in research investment across therapeutic areas. This scenario underscores the urgent need for greater incentives and funding to support the progression of promising compounds into robust preclinical and clinical development for NTDs.

Among the analyzed compounds, four candidates stood out due to their distinct structural profiles and mechanisms of action. Compound (**8**), a thiosemicarbazone derivative, contains a chlorine atom, which certainly influences its hydrophobic properties and probably also its interactions with biological targets. It exhibited significant activity against *T. cruzi* and cruzain, suggesting a potential enzyme inhibition mechanism. Compound (**79**) features a thiazole–triazole structure with a nitro group, a well-known pharmacophore for antiparasitic activity. It demonstrated potent effects against *T. b. rhodesiense* and *L. donovani*, showing promise for treating visceral leishmaniasis. Additionally, it exhibited a high SI, indicating a favorable safety profile. Compound (**81**), with a heterocyclic scaffold incorporating thiazole and substituted triazole groups, showed activity against *T. brucei*, *T. cruzi*, and *L. donovani*, along with promising pharmacokinetic properties. The compound (**133**) demonstrated strong antimalarial activity, significantly inhibiting the merozoite stage. Its structure, composed of a thiosemicarbazone core with condensed aromatic rings, suggests critical interactions with parasite targets. In the Supplementary Materials, we list a general overview of compounds by structural classification, LogP, biological targets, and evaluation in vitro (Table S1).

**Figure 17:**
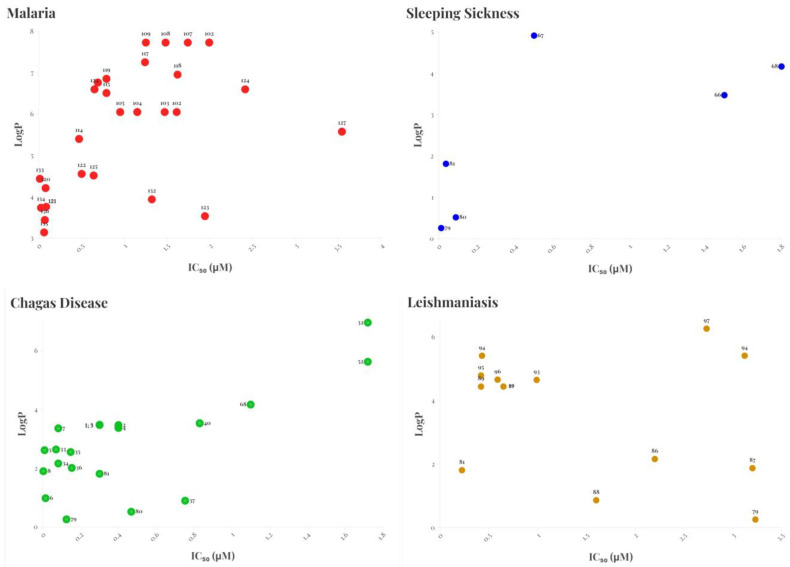
Relationship between hydrophobicity (LogP) and potency (IC_50_) of antiparasitic compounds.

The evaluated compounds target a wide range of therapeutic pathways, reflecting the biochemical diversity of NTD-causing parasites. For Chagas disease, the primary targets investigated were cruzipain and TcrPDEC. In sleeping sickness, research focused on *Tb*CatL and TR inhibition. In leishmaniasis, immunomodulatory compounds stood out, while for malaria, the main targets included DHFR and schizont maturation. Regarding pharmacokinetic properties, the compounds exhibited LogP values within the optimal range for absorption and permeability, balancing lipophilicity and solubility. The predominance of thiazole-based scaffolds suggests a structural profile favorable for effective interactions with biological targets (Figure 17).

Mechanistically, the reviewed compounds interfere with critical signaling pathways in parasites, including redox balance modulation (e.g., thiazole and derivatives), proteolytic activity (e.g., cruzipain [100] and cathepsin L inhibition [101]), folate biosynthesis (e.g., DHFR inhibitors [175]), and cyclic nucleotide regulation (e.g., TcrPDEC inhibitors [111]). These pathways are essential for parasite survival, replication, and host immune evasion, and thus represent strategic targets for therapeutic intervention.

The physicochemical and pharmacokinetic properties of the compounds described in this review article according to Lipinski’s rule, molecular weight, LogP, rotatable bonds, acceptor and donors, surface area, water solubility, Caco2 permeability, and intestinal absorption (human) were obtained by the online program pkCSM (Table S2), as shown in the Supplementary Materials [184].

These findings indicate that the identified compounds demonstrate promising antiparasitic activity against NTD pathogens and exhibit physicochemical properties compatible with desirable pharmacokinetic criteria. As a result, they represent viable candidates for further studies, including optimized structural modifications and detailed preclinical evaluations, aiming to develop effective therapeutic agents for NTD treatment.

## 3. Conclusions

The analysis of antiparasitic compounds described in the literature highlights the predominance of thiazoles and their analogs as key structural frameworks in the development of novel therapeutic agents for NTDs. The distribution of these compounds across pathogens suggests that these core structures exhibit preferential interactions with essential biological targets, including cruzain (*T. cruzi*), TR (*T. brucei*), DHFR (*Plasmodium* spp.), and proteins involved in schizont maturation. The presence of specific functional groups, such as thiosemicarbazones and conjugated heterocyclic systems, contributes to modulating biological activity and selectivity. Moreover, the evaluated pharmacokinetic properties suggest a favorable structural profile, which is crucial for the continued development of these compounds.

Despite the identification of promising molecules, the translation of these chemical entities into clinical candidates remains limited. The low number of in vivo studies and the lack of clinical trials targeting these diseases indicate a significant gap in advancing medicinal chemistry discoveries to later stages of pharmaceutical development. Furthermore, comparisons with other therapeutic areas reveal a stark imbalance in research funding allocated to NTDs. Addressing this disparity requires coordinated efforts to advance the pharmacological validation of promising compounds, including elucidation of their mechanisms of action, optimization of pharmacokinetic properties, and execution of robust preclinical studies. These findings not only highlight the promising pharmacological potential of thiazole derivatives but also underscore the need for systematic benchmarking against current therapies to accelerate their progression through the drug development pipeline.

## Data Availability

All the available data are reported in this work.

## Supplementary Materials

The following supporting information can be downloaded at https://www.mdpi.com/article/10.3390/molecules30081788/s1, Table S1: General overview of antiparasitic compounds: structural classification, LogP, biological targets, and evaluation in vitro; Table S2: Physico-chemical and pharmacokinetic properties of the compounds described in this review article according to Lipinski’s rule, molecular weight, cLogP, rotatable bonds, acceptor and donors, surface area, water solubility, Caco2 permeability, and intestinal absorption (human) by the online program pkCSM.

## Abbreviations

The following abbreviations are used in this manuscript:

ADMET	Absorption, distribution, metabolism, excretion, and toxicity
BBB	Blood–brain barrier
BZD	Benznidazole
CC_50_	Concentration of cytotoxicity 50%
CPB	Cysteine protease B
DAC	Diminazene aceturate
DHFR	Dihydrofolate reductase
DNA	Deoxyribonucleic acid
DNDi	Drugs for Neglected Diseases Initiative
EC_50_	Half maximal effective concentration
EpA	Oxidation potential
EpC	Reduction potential
FP2	Falcipain-2
HAT	Human African trypanosomiasis
IC_50_	Half maximal inhibitory concentration
IDO1	Indoleamine 2,3-dioxygenase 1
ITPK1	Inositol-tetrakisphosphate 1-kinase
Kd	Dissociation constant
Ki	Inhibition constant
*L.* spp.	*Leishmania* spp.
LC_50_	Lethal concentration 50%
LD_50_	Lethal dose 50%
LogP	Logarithm of the octanol–water partition coefficient
MTX	Methotrexate
NO	Nitric oxide
NTDs	Neglected tropical diseases
*P*. spp.	*Plasmodium* spp.
*Pf*FPPS/GGPPS	Farnesyl/geranylgeranyl diphosphate synthase
PTR1	Pteridine reductase 1
QSAR	Quantitative structure–activity relationship
ROS	Reactive oxygen species
RR	Ribonucleotide reductase
SAR	Structure–activity relationship
SEM	Scanning electron microscopy
SI	Selectivity index
*T. cruzi*	*Trypanosoma cruzi*
*T. b.* spp.	*Trypanosoma brucei* spp.
*Tb*CatL	*Trypanosoma brucei* cathepsin L
TcrPDEC	Phosphodiesterase C
TR	Trypanothione reductase
