# Supplementary material for: Recent Progress in Thiazole, Thiosemicarbazone, and Semicarbazone Derivatives as Antiparasitic Agents Against Trypanosomatids and Plasmodium spp
Source: Molecules. 2025 Apr 16;30(8):1788. doi: 10.3390/molecules30081788 (PMC12029465; doi:10.3390/molecules30081788)
Supplement: Supplementary file 1 [file molecules-30-01788-s001.zip › molecules-3575481-supplementary.pdf]

Recent Progress in Thiazole, Thiosemicarbazone, and Semicarbazone Derivatives as Antiparasitic Agents Against Trypanosomatids and *Plasmodium* spp.

Pamela Souza Tada da Cunha[1, #], Ana Luísa Rodriguez Gini[1, #], Chung Man Chin[1], Jean Leandro dos Santos[1] and Cauê Benito Scarim[1, *]

[1]Department of Drugs and Medicines, School of Pharmaceutical Sciences, Sao Paulo State University (UNESP), Araraquara, SP, Brazil; pamela.tada@unesp.br (P.S.T.d.C.); ana.gini@unesp.br (A.L.R.G); chung.man-chin@unesp.br (C.M.C.); jean.santos@unesp.br (J.L.d. S.); caue.scarim@unesp.br (C.B.S.)

*Correspondence: caue.scarim@unesp.br (C.B.S.)

#Both authors contributed equally.

Author information

Address: Rodovia Araraquara-Jaú km 1, s/n, Campus Ville, Zip Code 14800-903, Araraquara, São Paulo, Brazil. Phone: (+55)-16-33016971. Fax: (+55)-16-33016960. E-mail: caue.scarim@unesp.br (Scarim, CB).

Contents

Table S1 .... **3**
Table S2 .... **8**
References **13**

Table S1: General overview of antiparasitic compounds: structural classification, logP, biological targets and evaluation *in vitro*.

Compound	Category	LogP	Biological Target	Biological Activity (µM)	Measure
1	thiazole and analogues	3.4735	*T. cruzi*: amastigote and trypomastigote	0.3	IC_{50}
2	thiazole and analogues	3.4631	*T. cruzi*: amastigote and trypomastigote	0.4	IC_{50}
3	thiazole and analogues	3.4717	*T. cruzi*: amastigote and trypomastigote	0.3	IC_{50}
4	thiazole and analogues	3.3713	*T. cruzi*: amastigote and trypomastigote	0.4	IC_{50}
5	thiazole and analogues	2.61312	*T. cruzi*: not specified	0.31	EC_{50}
5	thiazole and analogues	2.61312	*T. cruzi*: cruzain	0.00899	IC_{50}
6	thiazole and analogues	0.9773	*T. cruzi*: not specified	0.77	EC_{50}
6	thiazole and analogues	0.9773	*T. cruzi*: cruzain	0.01431	IC_{50}
7	thiazole and analogues	3.35832	*T. cruzi*: not specified	0.53	EC_{50}
7	thiazole and analogues	3.35832	*T. cruzi*: cruzain	0.08169	IC_{50}
8	thiosemicarbazone and analogues	1.8972	*T. cruzi*: not specified	10.0	EC_{50}
8	thiosemicarbazone and analogues	1.8972	*T. cruzi*: cruzain	0.0025	IC_{50}
9	thiazole and analogues	8.6677	*T. cruzi*: epimastigote	2.8	IC_{50}
9	thiazole and analogues	8.6677	*T. cruzi*: trypomastigote	5.0	IC_{50}
10	thiazole and analogues	N/D	*T. cruzi*: trypomastigote	8.6	IC_{50}
11	thiazole and analogues	7.3695	*T. cruzi*: amastigote	1.9	IC_{50}
12	thiazole and analogues	3.2798	*T. cruzi*: amastigote	13.54	IC_{50}
13	thiazole and analogues	1.4634	*T. cruzi*: amastigote	10.37	IC_{50}
14	thiazole and analogues	5.123	*T. cruzi*: amastigote	3.9	IC_{50}
15	thiazole and analogues	3.1645	*T. cruzi*: amastigote	4.76	IC_{50}
16	thiazole and analogues	4.3691	*T. cruzi*: amastigote	7.25	IC_{50}
16	thiazole and analogues	4.3691	*L. amazonensis*: promastigote and amastigote	10.91	IC_{50}
16	thiazole and analogues	4.3691	*L. infantum*: promastigote and amastigote	8.47	IC_{50}
17	thiazole and analogues	4.3691	*L. amazonensis*: promastigote and amastigote	11.11	IC_{50}
17	thiazole and analogues	4.3691	*L. infantum*: promastigote and amastigote	7.86	IC_{50}
18	thiazole and analogues	2.7744	*L. amazonensis*: promastigote and amastigote	8.77	IC_{50}
18	thiazole and analogues	2.7744	*L. infantum*: promastigote and amastigote	11.07	IC_{50}
19	thiazole and analogues	4.2687	*L. amazonensis*: promastigote and amastigote	8.86	IC_{50}
19	thiazole and analogues	4.2687	*L. infantum*: promastigote and amastigote	6.43	IC_{50}

20	thiazole and analogues	2.1497	*T. cruzi*: epimastigote	6.0	IC_{50}
21	thiazole and analogues	3.1811	*T. cruzi*: epimastigote	14.0	IC_{50}
22	thiazole and analogues	2.8031	*T. cruzi*: epimastigote	16.0	IC_{50}
23	thiazole and analogues	3.6954	*T. cruzi*: epimastigote	16.0	IC_{50}
24	thiosemicarbazone and analogues	0.8709	*T. cruzi*: cruzain	9.0	IC_{50}
25	semicarbazone and analogues	0.706	N/D	N/D	N/D
26	thiazole and analogues	8.2734	*T. cruzi*: trypomastigote	2.54	IC_{50}
26	thiazole and analogues	8.2734	*T. cruzi*: epimastigote	8.62	IC_{50}
27	thiazole and analogues	7.9132	*T. cruzi*: trypomastigote	4.08	IC_{50}
27	thiazole and analogues	7.9132	*T. cruzi*: epimastigote	8.33	IC_{50}
28	thiazole and analogues	5.9616	*T. cruzi*: amastigote	3.65	IC_{50}
29	thiazole and analogues	7.2598	*T. cruzi*: trypomastigote	4.82	IC_{50}
30	thiazole and analogues	2.3213	*T. cruzi*: trypomastigote	25.0	IC_{50}
31	thiazole and analogues	6.9472	*T. cruzi*: trypomastigote	2.50	IC_{50}
31	thiazole and analogues	6.9472	*T. cruzi*: amastigote	6.12	IC_{50}
31	thiazole and analogues	6.9472	*L. amazonensis*: promastigote	19.86	IC_{50}
32	thiazole and analogues	6.9558	*T. cruzi*: trypomastigote	1.72	IC_{50}
32	thiazole and analogues	6.9558	*T. cruzi*: amastigote	1.96	IC_{50}
33	thiosemicarbazone and analogues	2.6375	*T. cruzi*: cruzipain	0.0695	IC_{50}
34	thiosemicarbazone and analogues	2.1605	*T. cruzi*: cruzipain	0.0816	IC_{50}
35	thiosemicarbazone and analogues	2.5506	*T. cruzi*: cruzipain	0.147	IC_{50}
36	thiosemicarbazone and analogues	2.0089	*T. cruzi*: cruzipain	0.153	IC_{50}
37	thiosemicarbazone and analogues	0.8907	*T. cruzi*: cruzipain	0.753	IC_{50}
38	thiosemicarbazone and analogues	2.7671	*T. cruzi*: trypomastigote	0.56	EC_{50}
39	thiosemicarbazone and analogues	3.5296	*T. cruzi*: trypomastigote	0.027	EC_{50}
40	thiazole and analogues	3.5283	*T. cruzi*: trypomastigote	0.83	IC_{50}
41	thiazole and analogues	2.3588	*T. cruzi*: trypomastigote	2.75	IC_{50}
42	thiazole and analogues	3.1559	*T. cruzi*: trypomastigote	2.83	IC_{50}
43	Others	1.86972	*L. amazonensis*: not specified	13.35	IC_{50}
43	Others	1.86972	*L. infantum*: not specified	18.82	IC_{50}
44	Others	2.3238	*L. amazonensis*: not specified	14.63	IC_{50}
44	Others	2.3238	*L. infantum*: not specified	14.49	IC_{50}
45	Others	5.6175	*T. cruzi*: trypomastigote	0.176	$LogIC_{50}$
46	Others	5.3586	*T. cruzi*: trypomastigote	-0.006	$LogIC_{50}$
47	Others	5.3814	*T. cruzi*: trypomastigote	0.397	$LogIC_{50}$

48	Others	5.1732	*T. cruzi*: trypomastigote	0.299	$LogIC_{50}$
49	thiazole and analogues	4.3762	*T. cruzi*: amastigote and trypomastigote	4.43	IC_{50}
50	thiazole and analogues	4.8581	*T. cruzi*: amastigote and trypomastigote	4.12	IC_{50}
50	thiazole and analogues	4.8581	*L. amazonensis*: promastigote	7.36	IC_{50}
50	thiazole and analogues	4.8581	*L. infantum*: promastigote	7.97	IC_{50}
51	thiazole and analogues	5.7748	*T. cruzi*: amastigote and trypomastigote	2.05	IC_{50}
52	thiazole and analogues	5.6206	*T. cruzi*: amastigote and trypomastigote	1.72	IC_{50}
52	thiazole and analogues	5.6206	*L. amazonensis*: promastigote	6.17	IC_{50}
52	thiazole and analogues	5.6206	*L. infantum*: promastigote	6.04	IC_{50}
53	Others	N/D	*T. cruzi*: trypomastigote	0.84	IC_{50}
54	Others	N/D	*T. cruzi*: trypomastigote	1.12	IC_{50}
55	Others	N/D	*T. cruzi*: trypomastigote	2.57	IC_{50}
56	Others	N/D	*T. cruzi*: trypomastigote	4.99	IC_{50}
57	thiazole and analogues	4.9821	*T. cruzi*: epimastigote	115.5	IC_{50}
58	thiazole and analogues	4.6803	*T. cruzi*: epimastigote	118.4	IC_{50}
58	thiazole and analogues	4.6803	*T. cruzi*: trypomastigote	363.1	IC_{50}
59	thiazole and analogues	4.6963	*T. cruzi*: epimastigote	23.1	IC_{50}
59	thiazole and analogues	4.6963	*T. cruzi*: trypomastigote	8.5	IC_{50}
60	thiazole and analogues	3.4679	*T. cruzi*: trypomastigote	3.6	IC_{50}
61	thiazole and analogues	3.0461	*T. cruzi*: trypomastigote	6.7	IC_{50}
62	thiazole and analogues	3.7731	*T. cruzi*: trypomastigote	10.2	IC_{50}
63	thiazole and analogues	3.3513	*T. cruzi*: trypomastigote	16.6	IC_{50}
64	thiosemicarbazone and analogues	1.4894	*T. cruzi*: not specified	24.1	IC_{50}
65	thiosemicarbazone and analogues	0.7092	*T. cruzi*: not specified	38.6	IC_{50}
66	thiazole and analogues	3.4679	*T. b. brucei*: not specified	1.5	IC_{50}
67	thiazole and analogues	4.9077	*T. b. brucei*: not specified	0.5	IC_{50}
68	thiazole and analogues	4.1606	*T. b. brucei*: not specified	1.8	IC_{50}
68	thiazole and analogues	4.1606	*T. cruzi*: not specified	1.1	IC_{50}
69	thiazole and analogues	4.91554	*T. b.*: not specified	0.00051	EC_{50}
70	thiazole and analogues	5.7314	*T. b.*: not specified	0.028	EC_{50}
71	thiazole and analogues	5.8272	*T. b.*: not specified	0.021	EC_{50}
72	thiazole and analogues	3.8972	*T. b.*: ITPK1	0.006	EC_{50}
73	thiazole and analogues	3.3548	*T. b.*: ITPK1	0.020	EC_{50}
74	thiazole and analogues	3.0908	*T. b.*: ITPK1	0.180	EC_{50}

75	thiazole and analogues	3.6609	*T. b.*: ITPK1	0.020	EC_{50}
76	thiazole and analogues	3.9791	*T. b.*: ITPK1	0.110	EC_{50}
77	thiazole and analogues	2.2109	N/D	N/D	N/D
78	thiazole and analogues	2.9205	N/D	N/D	N/D
79	thiazole and analogues	0.2564	*T. b. rhodesiense*: trypomastigote	0.012	IC_{50}
79	thiazole and analogues	0.2564	*T. cruzi*: amastigote	0.125	IC_{50}
79	thiazole and analogues	0.2564	*L. donovani*: amastigote	3.23	IC_{50}
80	thiazole and analogues	0.5153	*T. b. rhodesiense*: trypomastigote	0.089	IC_{50}
80	thiazole and analogues	0.5153	*T. cruzi*: amastigote	0.468	IC_{50}
81	thiazole and analogues	1.8092	*T. b. rhodesiense*: trypomastigote	0.037	IC_{50}
81	thiazole and analogues	1.8092	*T. cruzi*: amastigote	0.300	IC_{50}
81	thiazole and analogues	1.8092	*L. donovani*: amastigote	0.225	IC_{50}
82	thiazole and analogues	5.8231	*T. b.* not specified: trypomastigote	0.0118	EC_{50}
83	thiazole and analogues	3.9664	*T. b.* not specified: trypomastigote	0.0071	EC_{50}
84	thiazole and analogues	4.21882	*L. donovani*: not specified	10.07	IC_{50}
84	thiazole and analogues	4.21882	*T. cruzi*: not specified	55.48	IC_{50}
85	thiazole and analogues	3.1535	*L. amazonensis*: amastigote	7.074	IC_{50}
85	thiazole and analogues	3.1535	*L. amazonensis*: promastigote	28.86	IC_{50}
86	thiosemicarbazone and analogues	2.1605	*L. chagasi*: amastigote	2.2	IC_{50}
87	thiosemicarbazone and analogues	1.8725	*L. chagasi*: amastigote	3.2	IC_{50}
88	thiosemicarbazone and analogues	0.8623	*L. chagasi*: amastigote	1.6	IC_{50}
89	Others	4.4363	*L. infantum*: promastigote	0.42	IC_{50}
89	Others	4.4363	*L. infantum*: amastigote	0.65	IC_{50}
90	Others	4.806	*L. infantum*: promastigote	13.51	IC_{50}
90	Others	4.806	*L. infantum*: amastigote	15.23	IC_{50}
91	Others	2.0277	*L. infantum*: PTR1	44.16	EC_{50}
92	Others	2.0277	*L. brasiliensis*: PTR1	23.45	EC_{50}
93	thiazole and analogues	4.6462	*L. infantum*: promastigote	3.57	IC_{50}
93	thiazole and analogues	4.6462	*L. infantum*: amastigote	0.99	IC_{50}
94	thiazole and analogues	5.4087	*L. infantum*: promastigote	3.12	IC_{50}
94	thiazole and analogues	5.4087	*L. infantum*: amastigote	0.43	IC_{50}
95	thiazole and analogues	4.7853	*L. infantum*: promastigote	0.42	IC_{50}
96	thiazole and analogues	4.6548	*L. infantum*: promastigote	4.44	IC_{50}
96	thiazole and analogues	4.6548	*L. infantum*: amastigote	0.59	IC_{50}
97	thiazole and analogues	6.26142	*L. infantum*: promastigote	2.73	IC_{50}

98	thiosemicarbazone and analogues	5.54187	*T. cruzi*: epimastigote	25.7	IC_{50}
99	thiosemicarbazone and analogues	4.78797	*L. amazonensis*: promastigote	14.2	IC_{50}
99	thiosemicarbazone and analogues	4.78797	*T. cruzi*: epimastigote	19.1	IC_{50}
100	thiosemicarbazone and analogues	4.68757	*L. amazonensis*: promastigote	18.3	IC_{50}
100	thiosemicarbazone and analogues	4.68757	*T. cruzi*: epimastigote	24.1	IC_{50}
101	thiosemicarbazone and analogues	4.4877	*L. amazonensis*: promastigote	18.9	IC_{50}
101	thiosemicarbazone and analogues	4.4877	*T. cruzi*: epimastigote	30.3	IC_{50}
102	thiosemicarbazone and analogues	6.0501	*P. falciparum* (3D7): PfNDH2	1.61	IC_{50}
103	thiosemicarbazone and analogues	6.0501	*P. falciparum* (3D7): PfNDH2	1.47	IC_{50}
104	thiosemicarbazone and analogues	6.0501	*P. falciparum* (3D7): PfNDH2	1.15	IC_{50}
105	thiosemicarbazone and analogues	6.0501	*P. falciparum* (3D7): PfNDH2	0.95	IC_{50}
106	thiosemicarbazone and analogues	7.7321	*P. falciparum* (3D7): PfNDH2	1.99	IC_{50}
107	thiosemicarbazone and analogues	7.7321	*P. falciparum* (3D7): PfNDH2	1.74	IC_{50}
108	thiosemicarbazone and analogues	7.7321	*P. falciparum* (3D7): PfNDH2	1.48	IC_{50}
109	thiosemicarbazone and analogues	7.7321	*P. falciparum* (3D7): PfNDH2	1.25	IC_{50}
110	thiazole and analogues	4.0102	*P. falciparum* (Dd2): not specified	0.102	EC_{50}
111	thiazole and analogues	4.7298	*P.* não especificado: IDO1	23	IC_{50}
112	thiazole and analogues	2.7915	*P.* não especificado: IDO1	13	IC_{50}
113	thiazole and analogues	N/D	*P. falciparum*: not specified	0.53	IC_{50}
114	thiazole and analogues	5.3988	*P. falciparum*: not specified	0.47	IC_{50}
115	thiazole and analogues	6.51042	*P. falciparum*: not specified	0.79	IC_{50}
116	thiazole and analogues	6.7644	*P. falciparum*: not specified	0.69	IC_{50}
117	thiazole and analogues	7.25562	*P. falciparum* (3D7): não especificado	1.24	IC_{50}
118	thiazole and analogues	6.9558	*P. falciparum* (3D7): not specified	1.62	IC_{50}
119	thiazole and analogues	6.8554	*P. falciparum* (3D7): not specified	0.79	IC_{50}
120	Others	4.20852	*P.* not specified: DHFR	0.079	IC_{50}
121	Others	3.75444	*P.* not specified: DHFR	0.085	IC_{50}
122	thiazole and analogues	4.5525	*P. falciparum*: PfFPPS/GGPPS	0.5	IC_{50}
123	thiazole and analogues	3.5293	*P. falciparum*: PfFPPS/GGPPS	1.94	IC_{50}
124	Others	6.60032	*P.* não especificado: ßHF	0.65	IC_{50}
125	Others	4.51362	*P.* não especificado: ßHF	0.64	IC_{50}
126	thiazole and analogues	N/D	*P. falciparum* (3D7): schizont	1.24	IC_{50}
127	thiazole and analogues	N/D	*P. falciparum* (3D7): schizont	1.78	IC_{50}
128	thiazole and analogues	N/D	*P. falciparum* (3D7): schizont	2.41	IC_{50}

129	semicarbazone and analogues	4.9803	*T. cruzi* (Y): trypomastigote	3.75	IC_{50}
130	semicarbazone and analogues	3.8126	*T. cruzi* (Y): trypomastigote	3.60	IC_{50}
131	thiazole and analogues	5.5759	*P. falciparum* (3D7): schizont	3.54	IC_{50}
131	thiazole and analogues	5.5759	*T. cruzi* (Y): trypomastigote	4.48	IC_{50}
132	thiazole and analogues	3.9355	*P. falciparum* (3D7): schizont	1.32	IC_{50}
133	thiosemicarbazone and analogues	4.4346	*P. falciparum*: FP2	0.0121	IC_{50}
134	thiosemicarbazone and analogues	3.7324	*P. falciparum*: FP2	0.0255	IC_{50}
135	thiosemicarbazone and analogues	3.1364	*P. falciparum*: FP2	0.0619	IC_{50}
136	thiosemicarbazone and analogues	3.43622	*P. falciparum*: FP2	0.0699	IC_{50}
137	thiosemicarbazone and analogues	1.3829	*P. falciparum* (3D7): schizont	13.54	EC_{50}
138	thiosemicarbazone and analogues	2.1963	*P. falciparum* (3D7): schizont	15.83	EC_{50}
139	thiosemicarbazone and analogues	0.8795	*P. falciparum* (3D7): schizont	14.52	EC_{50}

Table S2. Physico-chemical and pharmacokinetic properties of the compounds described in this article review according to Lipinski's rule, molecular weight, cLog*P*, rotatable bonds, acceptor and donors, surface area, water solubility, Caco2 permeability and intestinal absorption (human) by the on-line program pkCSM.

Compound	Molecular Weight	cLogP	Rotatable Bonds	H bond Acceptors	H bond Donors	Surface Area	Water solubility (log mol/L)	Caco2 permeability (log Papp in 10-6 cm/s)	Intestinal absorption (% absorbed)	Reference
1	308.41	3.4735	3	5	0	132.691	-4.164	1.637	98.131	[1]
2	294.383	34631	3	4	1	126.116	-4.221	1.396	94.679	[1]
3	324.409	3.4717	4	5	1	137.594	-4.42	0.966	95.622	[1]
4	339.38	3.3713	4	6	1	140.769	-4.425	0.309	94.584	[1]
5	359.675	2.61312	4	5	1	127.782	-4.527	0.879	92.039	[2]
6	319.064	0.9773	3	3	2	99.248	-2.244	1.229	77.329	[2]
7	349.123	3.35832	3	3	1	123.432	-4.532	1.738	92.261	[2]
8	227.72	1.8972	2	2	2	92.943	-3.187	1.253	91.672	[2]
9	639.402	8.6677	8	6	0	248.287	-6.12	1.017	92.68	[3]
11	600.538	7.3695	9	7	0	239.158	-5.684	1.039	95.311	[3]
12	312.185	3.2798	2	6	1	122.147	-3.233	1.242	93.962	[4]
13	303.307	1.4634	3	9	2	121.534	-2.832	0.698	78.763	[4]
14	444.354	5.123	7	6	0	169.998	-5.357	1.009	93.839	[5]
15	319.386	3.1645	6	7	1	132.342	-4.143	1.06	93.132	[5]
16	395.484	4.3691	8	7	0	167.609	-5.214	0.596	96.253	[5]
17	395.484	4.3691	8	7	0	167.609	-5.095	0.527	96.179	[5]
18	305.359	2.7744	6	7	1	125.977	-3.885	0.919	93.244	[5]
19	410.455	4.2687	8	8	0	170.784	-5.182	0.911	100	[5]
20	315.358	2.1497	4	8	2	129.911	-2.732	1.482	84.956	[6]
21	346.309	3.1811	2	6	1	131.437	-4.802	1.385	91.219	[6]
22	349.803	2.8031	4	8	1	140.214	-2.935	1.325	86.1	[6]
23	362.764	3.6954	2	6	1	137.575	-5.181	1.417	90.317	[6]
24	239.3	0.8709	4	4	2	99.232	-2.543	0.59	75.123	[7]
25	223.232	0.706	4	4	2	93.035	-2.164	0.635	68.752	[7]
26	472.828	8.2734	5	4	1	193.518	-6.951	1.006	87.784	[8]
27	465.62	7.9132	4	4	1	179.067	-7.393	1.004	84.577	[8]
28	392.311	5.9616	5	5	1	159.636	-5.866	1.031	88.912	[8]
29	431.175	7.2598	4	4	1	168.764	-6.908	1.026	86.301	[8]
30	403.179	2.3213	0	5	1	126.128	-3.573	1.322	91.726	[9]
31	439.971	6.9472	4	4	0	188.783	-7.715	1.025	93.784	[10]
32	469.997	6.9558	5	5	0	200.262	-7.438	1.016	93.864	[10]

33	276.192	2.6375	4	2	2	109.933	-3.615	1.492	88.215	[11]
34	248.138	2.1605	2	2	2	96.882	-3.297	1.301	89.666	[11]
35	262.165	2.5506	2	2	2	103.247	-3.828	1.334	89.506	[11]
36	286.198	2.0089	4	2	2	102.873	-3.108	1.279	91.897	[11]
37	252.299	0.8907	5	4	2	103.658	-3.196	0.015	74.294	[11]
38	279.752	2.7671	3	3	2	114.430	-3.755	0.911	91.792	[12]
39	358.648	3.5296	3	3	2	128.298	-4.798	0.834	91.041	[12]
40	354.229	3.5283	5	6	1	128.366	-4.174	0.825	90.422	[13]
41	265.294	2.3588	5	7	1	107.293	-3.628	0.927	93.526	[13]
42	289.36	3.1559	5	6	1	120.864	-3.647	0.898	91.997	[13]
43	319.386	1.86972	5	6	1	132.580	-3.298	1.011	94.228	[13]
44	384.255	2.3238	5	6	1	140.083	-3.884	0.985	92.703	[13]
45	345.486	5.6175	5	1	0	156.404	-5.534	1.08	91.859	[14]
46	303.449	5.3586	5	1	1	139.303	-4.79	1.679	91.018	[14]
47	317.432	5.3814	5	2	1	143.464	-5.594	1.687	92.648	[14]
48	319.448	5.1732	5	2	2	144.097	-5.243	1.672	90.123	[14]
49	443.488	4.3762	5	7	2	187.332	-4.388	0.312	98.227	[15]
50	412.518	4.8581	4	5	2	179.044	-4.578	0.964	89.53	[15]
51	467.381	5.7748	4	5	2	193.285	-4.781	0.977	87.749	[15]
52	491.414	5.6206	4	5	2	192.911	-4.699	0.951	88.493	[15]
57	279.286	4.9821	1	2	0	110.892	-5.581	1.725	92.901	[16]
58	323.295	4.6803	2	3	1	126.212	-4.199	1.412	91.946	[16]
59	325.311	4.6963	2	4	1	127.164	-5.041	1.379	91.277	[17]
60	317.805	3.4679	4	6	1	131.747	-4.276	0.864	92.999	[18]
61	281.344	3.0461	4	6	1	119.594	-3.842	0.871	94.037	[18]
62	322.796	3.7731	4	5	1	131.047	-3.932	1.341	93.31	[18]
63	286.335	3.3513	4	5	1	118.894	-3.583	1.308	94.348	[18]
64	357.443	1.4894	6	6	3	150.023	-4.078	0.145	74.723	[19]
65	329.389	0.7092	4	6	3	137.293	-3.901	0.13	73.272	[19]
66	320.167	3.4679	1	4	0	115.303	-3.796	1.426	94.602	[20]
67	411.28	4.9077	3	5	1	155.910	-4.7	1.03	89.46	[20]
68	363.394	4.1606	4	6	0	153.085	-4.546	1.198	96.837	[20]
69	434.594	4.91554	4	6	4	181.536	-4.129	0.603	91.565	[21]
70	516.74	5.7314	9	8	2	219.560	-5.76	0.588	92.124	[21]
71	514.724	5.8272	4	8	2	218.554	-5.383	0.591	90.762	[21]
72	381.326	3.8972	6	6	2	142.166	-4.497	1.022	88.78	[22]
73	327.356	3.3548	6	6	2	129.669	-3.866	1.016	91.295	[22]
74	327.356	3.0908	5	6	3	129.350	-3.421	1.031	92.29	[22]
75	388.39	3.6609	5	6	2	150.371	-4.182	0.989	90.539	[22]

76	337.395	3.9791	4	5	2	136.280	-4.554	0.975	88.835	[22]
77	305.384	2.2109	2	6	2	122.675	-3.679	0.901	90.502	[23]
78	302.399	2.9205	5	4	1	128.430	-3.554	1.303	93.246	[23]
79	295.328	0.2564	3	9	1	117.957	-2.266	0.314	77.903	[24]
80	337.365	0.5153	3	9	0	135.058	-2.313	0.317	76.798	[24]
81	399.436	1.8092	4	9	0	163.750	-2.78	0.693	82.598	[24]
82	590.728	5.8231	8	11	3	240.047	-4.474	-0.404	73.806	[25]
83	520.593	3.9664	7	11	3	208.222	-3.756	-0.202	69.994	[25]
84	450.48	4.21882	8	10	1	186.227	-3.152	0.31	100	[26]
85	285.328	3.1535	3	6	3	118.399	-3.431	0.917	86.875	[27]
86	248.138	2.1605	2	2	2	96.882	-3.297	1.301	89.666	[28]
87	247.245	1.8725	2	2	2	95.137	-3.356	1.302	89.715	[28]
88	209.274	0.8623	3	3	2	87.754	-2.218	1.205	92.112	[28]
89	451.932	4.4363	5	6	1	180.164	-6.049	0.59	88.389	[29]
90	436.961	4.806	5	5	1	176.990	-5.813	1.316	89.034	[29]
91	265.29	2.0277	3	5	1	108.149	-2.889	1.016	94.016	[30]
92	265.29	2.0277	3	5	1	108.149	-2.888	0.957	94.364	[30]
93	293.395	4.6462	4	4	1	127.551	-4.733	1.696	91.113	[31]
94	372.291	5.4087	4	4	1	141.418	-5.404	1.548	89.597	[31]
95	311.385	4.7853	4	4	1	131.716	-4.732	1.486	90.733	[31]
96	323.421	4.6548	5	5	1	139.029	-4.733	1.414	92.141	[31]
97	376.312	6.26142	4	4	1	154.522	-6.237	1.019	88.151	[31]
98	482.329	5.54187	4	5	2	177.201	-4.988	0.625	87.723	[32]
99	433.459	4.78797	5	6	2	174.811	-5.396	0.661	88.136	[32]
100	448.43	4.68757	5	7	2	177.986	-5.111	0.544	91.254	[32]
101	468.302	4.4877	4	4	2	171.726	-4.379	0.658	89.926	[32]
102	819.775	6.0501	14	12	2	317.387	-3.53	0.637	69.6	[33]
103	819.535	6.0501	14	12	2	317.267	-3.53	0.637	69.626	[33]
104	824.388	6.0501	14	12	2	317.508	-3.529	0.637	69.575	[33]
105	826.232	6.0501	14	12	2	318.510	-3.528	0.637	69.364	[33]
106	917.515	7.7321	12	10	4	321.527	-3.78	0.402	77.247	[33]
107	917.275	7.7321	12	10	4	321.407	-3.78	0.402	77.272	[33]
108	922.128	7.7321	12	10	4	321.647	-3.78	0.402	77.221	[33]
109	923.972	7.7321	12	10	4	322.649	-3.778	0.402	77.01	[33]
110	635.794	4.0102	12	7	4	271.171	-3.079	0.31	71.028	[34]
111	276.364	4.7298	2	3	0	120.524	-2.968	1.733	92.13	[34]
112	244.275	2.7915	1	5	0	101.053	-2.821	1.559	94.491	[34]
114	414.49	5.3988	6	6	0	176.481	-6.494	0.369	93.122	[35]
115	464.55	6.51042	5	6	0	199.498	-7.275	0.242	93.973	[35]

116	478.577	6.7644	6	6	0	205.863	-7.187	0.221	94.417	[35]
117	453.998	7.25562	4	4	0	195.148	-7.579	1.077	94.418	[36]
118	469.997	6.9558	5	5	0	200.262	-7.524	1.043	94.924	[36]
119	484.968	6.8554	5	6	0	203.436	-7.21	0.179	92.095	[36]
120	455.337	4.20852	3	5	2	172.879	-2.954	0.795	88.437	[37]
121	390.468	3.75444	3	5	2	165.376	-2.951	0.992	89.962	[37]
122	391.512	4.5525	7	5	1	163.107	-4.61	1.192	90.288	[38]
123	381.448	3.5293	6	5	1	154.543	-4.3	1.301	92.104	[38]
124	561.088	6.60032	7	7	2	231.757	-4.968	0.597	100	[39]
125	484.99	4.51362	5	7	3	196.380	-4.323	0.559	86.772	[39]
129	483.38	4.9803	5	4	2	199.378	-4.393	0.723	87.353	[40]
130	432.48	3.8126	5	4	2	182.937	-3.834	0.863	90.452	[40]
131	472.957	5.5759	6	6	1	198.718	-4.581	0.632	90.152	[40]
132	275.483	3.9355	10	4	1	114.049	-4.97	1.464	90.583	[41]
133	398.706	4.4346	2	3	3	157.383	-2.889	1.059	77.473	[42,43]
134	455.712	3.7324	2	3	3	156.038	-2.888	0.756	81.883	[42,43]
135	359.842	3.1364	3	4	3	148.254	-2.891	0.817	83.014	[42]
136	343.843	3.43622	2	3	3	143.141	-2.888	0.775	82.773	[42]
137	211.265	1.3829	2	2	2	86.806	-2.668	1.21	92.038	[44]
138	272.171	2.1963	2	2	2	96.508	-3.314	1.259	91.605	[44]
139	269.326	0.8795	5	5	2	110.711	-2.874	0.552	85.251	[44]

References

[1] V. Gouveia de Melo Silva, L. Manoel da Silva Sousa, E.L. Fernandes Junior, G.L. Brondani, I. Maria de Albuquerque Oliveira, D.C. Galindo Bedor, I.B. Pereira Lopes, F.A. Brayner, L.C. Alves, M. Kaique de Andrade Cavalcante, D. Santana de Souza Oliveira, M.C. Accioly Brelaz-de-Castro, P.A. Sales Junior, V.R. Alves Pereira, A.C. Lima Leite, New series of 3-pyridyl-1,3-thiazoles: In vitro and in vivo anti-Trypanosomatidae profile, in vitro and in silico mechanism of action approach, Eur J Med Chem 284 (2025) 117191. https://doi.org/10.1016/j.ejmech.2024.117191.

[2] M. Rubio-Hernández, V. Alcolea, E. Barbosa da Silva, M.A. Giardini, T.H. M Fernandes, N. Martínez-Sáez, A.J. O'Donoghue, J.L. Siqueira-Neto, S. Pérez-Silanes, Synthesis and Biological Evaluation of New Chalcogen Semicarbazone (S, Se) and Their Azole Derivatives against Chagas Disease, J Med Chem 67 (2024) 19038–19056. https://doi.org/10.1021/acs.jmedchem.4c01535.

[3] A.C. Cristovão-Silva, M.C.A. Brelaz-de-Castro, E. Dionisio da Silva, A.C.L. Leite, L.B.A.A. Santiago, J.M. da Conceição, R. da Silva Tiburcio, D.P. de Santana, D.C.G. Bedor, B.Í.V. de Carvalho, L.F.G.R. Ferreira, R. de Freitas e Silva, V.R. Alves Pereira, M.Z. Hernandes, Trypanosoma cruzi killing and immune response boosting by novel phenoxyhydrazine-thiazole against Chagas disease, Exp Parasitol 261 (2024). https://doi.org/10.1016/j.exppara.2024.108749.

[4] T.P. de Souza, L.M.R. Orlando, L. da S. Lara, V.B. Paes, L.P. Dutra, M.S. dos Santos, M.C. de S. Pereira, Synthesis and Anti-Trypanosoma cruzi Activity of New Pyrazole-Thiadiazole Scaffolds, Molecules 29 (2024). https://doi.org/10.3390/molecules29153544.

[5] M. Haroon, T. Akhtar, H. Mehmood, A.C. da Silva Santos, J.M. da Conceição, G.L. Brondani, R. da Silva Tibúrcio, D.C. Galindo Bedor, J.W. Viturino da Silva, P.A. Sales Junior, V.R. Alves Pereira, A.C. Lima Leite, Synthesis of hydrazinyl–thiazole ester derivatives, in vitro trypanocidal and leishmanicidal activities, Future Med Chem 16 (2024) 221–238. https://doi.org/10.4155/fmc-2023-0255.

[6] A.F.M. Faria, C. de Souza Ferreira Pereira, G.P. Teixeira, R.M. dos Santos Galvão, P.A.F. Pacheco, M.L. Bello, D.H. de Jesus, K. Calabrese, D.T.G. Gonzaga, N. Boechat, R.X. Faria, In vitro evaluation of 2-(1H-pyrazol-1-yl)-1,3,4-thiadiazole derivatives against replicative and infective stages of Trypanosoma cruzi, J Bioenerg Biomembr 55 (2023) 409–421. https://doi.org/10.1007/s10863-023-09982-7.

[7] L.C. Martins, R.B. de Oliveira, J. Lameira, R.S. Ferreira, Experimental and Computational Study of Aryl-thiosemicarbazones Inhibiting Cruzain Reveals Reversible Inhibition and a Stepwise Mechanism, J Chem Inf Model 63 (2023) 1506–1520. https://doi.org/10.1021/acs.jcim.2c01566.

[8] M. Cox Holanda de Barros Dias, M. Souza Barbalho, G. Bezerra de Oliveira Filho, M. Veríssimo de Oliveira Cardoso, A.C. Lima Leite, A.C. da Silva Santos, A.C. Cristovão Silva, M.C. Accioly Brelaz de Castro, D. Maria Nascimento Moura, L.F. Gomes Rebello Ferreira, M. Zaldini Hernandes, R. de Freitas e Silva, V. Rêgo Alves Pereira, 1,3-Thiazole derivatives as privileged structures for anti-Trypanosoma cruzi activity: Rational design, synthesis, in silico and in vitro studies, Eur J Med Chem 257 (2023) 115508. https://doi.org/10.1016/j.ejmech.2023.115508.

[9] S. Rostán, S. Porto, C.L.N. Barbosa, D. Assis, N. Alvarez, F.S. Machado, G. Mahler, L. Otero, A novel palladium complex with a coumarin-thiosemicarbazone hybrid ligand inhibits Trypanosoma cruzi release from host cells and lowers the parasitemia in vivo, Journal of Biological Inorganic Chemistry 28 (2023) 711–723. https://doi.org/10.1007/s00775-023-02020-2.

[10] I.J. Da Cruz Filho, J.F. De Oliveira, A.C.S. Santos, V.R.A. Pereira, M.C.A. De Lima, Synthesis of 4-(4-chlorophenyl)thiazole compounds: in silico and in vitro evaluations as leishmanicidal and trypanocidal agents, An Acad Bras Cienc 95 (2023). https://doi.org/10.1590/0001-3765202320220538.

[11] G. Jasinski, E. Salas-Sarduy, D. Vega, L. Fabian, M.F. Martini, A.G. Moglioni, Thiosemicarbazone derivatives: Evaluation as cruzipain inhibitors and molecular modeling study of complexes with cruzain, Bioorg Med Chem 61 (2022). https://doi.org/10.1016/j.bmc.2022.116708.

[12] S.F.P. Braga, V.C. Santos, R.P. Vieira, E.B. da Silva, L. Monti, S.H. Krake, P.D.G. Martinez, L.C. Dias, C.R. Caffrey, J.L. Siqueira-Neto, R.B. de Oliveira, R.S. Ferreira, From rational design to serendipity: Discovery of novel thiosemicarbazones as potent trypanocidal compounds, Eur J Med Chem 244 (2022) 114876. https://doi.org/10.1016/j.ejmech.2022.114876.

[13] M. Haroon, M.C.H. De Barros Dias, A.C. da S. Santos, V.R.A. Pereira, L.A.B. Freitas, R.B. Balbinot, V. Kaplum, C.V. Nakamura, L.C. Alves, F.A. Brayner, A.C.L. Leite, T. Akhtar, The design, synthesis, and: In vitro trypanocidal and leishmanicidal activities of 1,3-thiazole and 4-thiazolidinone ester derivatives, RSC Adv 11 (2021) 2487–2500. https://doi.org/10.1039/d0ra06994a.

[14] D.L. Nossa González, J.A. Gómez Castaño, W.E. Rozo Núñez, P.R. Duchowicz, Antiprotozoal QSAR modelling for trypanosomiasis (Chagas disease) based on thiosemicarbazone and thiazole derivatives, J Mol Graph Model 103 (2021) 107821. https://doi.org/10.1016/j.jmgm.2020.107821.

[15] L.A. Barros Freitas, A. Caroline da Silva Santos, G. de Cássia Silva, F. Nayara do Nascimento Albuquerque, E.D. Silva, C. Alberto de Simone, V.R. Alves Pereira, L.C. Alves, F.A. Brayner, A.C. Lima Leite, P.A.T. de Moraes Gomes, Structural improvement of new thiazolyl-isatin derivatives produces potent and selective trypanocidal and leishmanicidal compounds, Chem Biol Interact 345 (2021) 109561. https://doi.org/10.1016/j.cbi.2021.109561.

[16] S. Martínez-Cerón, N. Andrea Gutiérrez-Nágera, E. Mirzaeicheshmeh, R.I. Cuevas-Hernández, J.G. Trujillo-Ferrara, Phenylbenzothiazole derivatives: effects against a Trypanosoma cruzi infection and toxicological profiles, (n.d.). https://doi.org/10.1007/s00436-021-07137-4/Published.

[17] R.I. Cuevas-Hernández, R.M.B.M. Girard, S. Martínez-Cerón, M. Santos da Silva, M.C. Elias, M. Crispim, J.G. Trujillo-Ferrara, A.M. Silber, A Fluorinated Phenylbenzothiazole Arrests the Trypanosoma cruzi Cell Cycle and Diminishes the Infection of Mammalian Host Cells, Antimicrob Agents Chemother 64 (2020). https://doi.org/10.1128/AAC.01742-19.

[18] R.H.C.N. Freitas, J.M.C. Barbosa, P. Bernardino, V. Sueth-Santiago, S.M.S.V. Wardell, J.L. Wardell, D. Decoté-Ricardo, T.G. Melo, E.F. da Silva, K. Salomão, C.A.M. Fraga, Synthesis and trypanocidal activity of novel pyridinyl-1,3,4-thiadiazole derivatives, Biomedicine and Pharmacotherapy 127 (2020). https://doi.org/10.1016/j.biopha.2020.110162.

[19] B.N.M. Silva, P.A. Sales Junior, A.J. Romanha, S.M.F. Murta, C.H.S. Lima, M.G. Albuquerque, E. D'Elia, J.G.A. Rodrigues, V.F. Ferreira, F.C. Silva, A.C. Pinto, B. V. Silva, Synthesis of New Thiosemicarbazones and Semicarbazones Containing the 1,2,3-1H-triazole-isatin Scaffold: Trypanocidal, Cytotoxicity, Electrochemical Assays, and Molecular Docking, Med Chem (Los Angeles) 15 (2018) 240–256. https://doi.org/10.2174/1573406414666180912120502.

[20] A. Ballesteros-Casallas, C. Quiroga, C. Ortiz, D. Benítez, P.A. Denis, D. Figueroa, C.O. Salas, J. Bertrand, R.A. Tapia, P. Sánchez, G. Pietro Miscione, M.A. Comini, M. Paulino, Mode of action of p-quinone derivatives with trypanocidal activity studied by experimental and in silico models, Eur J Med Chem 246 (2023). https://doi.org/10.1016/j.ejmech.2022.114926.

[21] L. Racané, L. Ptiček, S. Kostrun, S. Raić-Malić, M.C. Taylor, M. Delves, S. Alsford, F. Olmo, A.F. Francisco, J.M. Kelly, Bis-6-amidino-benzothiazole Derivative that Cures Experimental Stage 1 African Trypanosomiasis with a Single Dose, J Med Chem 66 (2023) 13043–13057. https://doi.org/10.1021/acs.jmedchem.3c01051.

[22] L.A.T. Cleghorn, R.J. Wall, S. Albrecht, S.A. MacGowan, S. Norval, M. De Rycker, A. Woodland, D. Spinks, S. Thompson, S. Patterson, V. Corpas Lopez, G. Dey, I.T. Collie, I. Hallyburton, R. Kime, F.R.C. Simeons, L. Stojanovski, J.A. Frearson, P.G. Wyatt, K.D. Read, I.H. Gilbert, S. Wyllie,

Development of a 2,4-Diaminothiazole Series for the Treatment of Human African Trypanosomiasis Highlights the Importance of Static-Cidal Screening of Analogues, J Med Chem 66 (2023) 8896–8916. https://doi.org/10.1021/acs.jmedchem.3c00509.

[23] S. Hendrickx, D. Bulté, D. Mabille, R. Mols, M. Claes, K. Ilbeigi, R. Ahmad, L. Dirkx, S.I. Van Acker, G. Caljon, Comparison of Bioluminescent Substrates in Natural Infection Models of Neglected Parasitic Diseases, Int J Mol Sci 23 (2022) 16074. https://doi.org/10.3390/ijms232416074.

[24] A. Mousavi, P. Foroumadi, Z. Emamgholipour, P. Mäser, M. Kaiser, A. Foroumadi, 2-(Nitroaryl)-5-Substituted-1,3,4-Thiadiazole Derivatives with Antiprotozoal Activities: In Vitro and In Vivo Study, Molecules 27 (2022) 5559. https://doi.org/10.3390/molecules27175559.

[25] J. Franco, L. Scarone, M.A. Comini, Novel distamycin analogues that block the cell cycle of African trypanosomes with high selectivity and potency, Eur J Med Chem 189 (2020) 112043. https://doi.org/10.1016/j.ejmech.2020.112043.

[26] A. Mijoba, N. Parra-Giménez, E. Fernandez-Moreira, H. Ramírez, X. Serrano, Z. Blanco, S. Espinosa, J.E. Charris, Synthesis of Hybrid Molecules with Imidazole-1,3,4-thiadiazole Core and Evaluation of Biological Activity on Trypanosoma cruzi and Leishmania donovani, Molecules 29 (2024) 4125. https://doi.org/10.3390/molecules29174125.

[27] E.S. Coimbra, L.M.R. Antinarelli, A.S. de Oliveira Lemos, A.F. da Silva Neto, A.C. Pinheiro, M.V.N. de Souza, Synthesis, biological evaluation and mechanism of action of benzothiazole derivatives with aromatic hydrazone moiety, a new class of antileishmanial compounds, Chem Biol Drug Des 104 (2024). https://doi.org/10.1111/cbdd.14585.

[28] T.M. de Aquino, P.H.B. França, É.E.E.S. Rodrigues, Igor.J.S. Nascimento, P.F.S. Santos-Júnior, P.G. V. Aquino, M.S. Santos, A.C. Queiroz, M. V. Araújo, M.S. Alexandre-Moreira, R.R.L. Rodrigues, K.A.F. Rodrigues, J.D. Freitas, J. Bricard, M.R. Meneghetti, J.-J. Bourguignon, M. Schmitt, E.F. da Silva-Júnior, J.X. de Araújo-Júnior, Synthesis, Antileishmanial Activity and in silico Studies of Aminoguanidine Hydrazones (AGH) and Thiosemicarbazones (TSC) Against Leishmania chagasi Amastigotes, Med Chem (Los Angeles) 18 (2021) 151–169. https://doi.org/10.2174/1573406417666210216154428.

[29] A.L.A. Gouveia, F.A.B. Santos, L.C. Alves, I.J. Cruz-Filho, P.R. Silva, I.T.T. Jacob, J.C.S. Soares, D.K.D.N. Santos, T.R.C.L. Souza, J.F. Oliveira, M. do C.A. Lima, Thiazolidine derivatives: In vitro toxicity assessment against promastigote and amastigote forms of Leishmania infantum and ultrastructural study, Exp Parasitol 236–237 (2022). https://doi.org/10.1016/j.exppara.2022.108253.

[30] F.S.M. Neri, D.B.C. Júnior, T.Q. Froes, P.B.G. da Silva, M.S. do Egito, P.O.L. Moreira, F. de Pilla Varotti, M.S. Castilho, R.G. Teixeira-Neto, J.F.C. de Albuquerque, F.H.A. Leite, Antileishmanial activity evaluation of thiazolidine-2,4-dione against Leishmania infantum and Leishmania braziliensis, Parasitol Res 119 (2020) 2263–2274. https://doi.org/10.1007/s00436-020-06706-3.

[31] V.V.G. de Oliveira, M.A. Aranda de Souza, R.R.M. Cavalcanti, M.V. de Oliveira Cardoso, A.C.L. Leite, V.A. da Silva Junior, R.C.B.Q. de Figueiredo, Study of in vitro biological activity of thiazoles on Leishmania (Leishmania) infantum, J Glob Antimicrob Resist 22 (2020) 414–421. https://doi.org/10.1016/j.jgar.2020.02.028.

[32] J. do N.A. Camargo, K.E. Pianoski, M.G. dos Santos, D. Lazarin-Bidóia, H. Volpato, S. Moura, C.V. Nakamura, F.A. Rosa, Antiparasitic Behavior of Trifluoromethylated Pyrazole 2-Amino-1,3,4-thiadiazole Hybrids and Their Analogues: Synthesis and Structure-Activity Relationship, Front Pharmacol 11 (2020). https://doi.org/10.3389/fphar.2020.591570.

[33] B. Kumar, J. Devi, A. Dubey, A. Tufail, S. Sharma, Exploring the antimalarial, antioxidant, anti-inflammatory activities of newly synthesized transition metal(II) complexes bearing thiosemicarbazone

ligands: Insights from molecular docking, DFT, MESP and ADMET studies, Inorg Chem Commun 159 (2024) 111674. https://doi.org/10.1016/j.inoche.2023.111674.

[34] R. Rayala, P. Chaudhari, A. Bunnell, B. Roberts, D. Chakrabarti, A. Nefzi, Parallel Synthesis of Piperazine Tethered Thiazole Compounds with Antiplasmodial Activity, Int J Mol Sci 24 (2023) 17414. https://doi.org/10.3390/ijms242417414.

[35] N. de F.N. Santos, N. da S.B. Junior, J.F. de Oliveira, D.M.F.A. Duarte, J.C. dos Santos Soares, D.S. Clara Marques, A.C. da Silva Santos, F. Nogueira, V.R. Alves Pereira, M.C. Alves de Lima, I.J. da Cruz Filho, Synthesis, characterization, antioxidant and antiparasitic activities new naphthyl-thiazole derivatives, Exp Parasitol 248 (2023) 108498. https://doi.org/10.1016/j.exppara.2023.108498.

[36] B.R.M.G. DA SILVA, N.D.S. BEZERRA JÚNIOR, J.F. DE OLIVEIRA, D.M.F.A. DUARTE, D.S.C. MARQUES, F. NOGUEIRA, M.C.A. DE LIMA, I.J. DA CRUZ FILHO, In silico ADMET prediction, evaluation of cytotoxicity in mouse splenocytes and preliminary evaluation of in vitro antimalarial activity of 4-(4-chlorophenyl)thiazole compounds, An Acad Bras Cienc 95 (2023). https://doi.org/10.1590/0001-3765202320230566.

[37] M.A. Ewida, H.A. Ewida, M.S. Ahmed, H.A. Allam, R.I. ElBagary, R.F. George, H.H. Georgey, H.I. El-Subbagh, 3-Methyl-imidazo[2,1-b]thiazole derivatives as a new class of antifolates: Synthesis, in vitro/in vivo bio-evaluation and molecular modeling simulations, Bioorg Chem 115 (2021) 105205. https://doi.org/10.1016/j.bioorg.2021.105205.

[38] S. Kabeche, J. Aida, T. Akther, T. Ichikawa, A. Ochida, M.J. Pulkoski-Gross, M. Smith, P.S. Humphries, E. Yeh, Nonbisphosphonate inhibitors of Plasmodium falciparum FPPS/GGPPS, Bioorg Med Chem Lett 41 (2021) 127978. https://doi.org/10.1016/j.bmcl.2021.127978.

[39] H. Ramírez, E. Fernandez, J. Rodrigues, S. Mayora, G. Martínez, C. Celis, J.B. De Sanctis, M. Mijares, J. Charris, Synthesis and antimalarial and anticancer evaluation of 7-chlorquinoline-4-thiazoleacetic derivatives containing aryl hydrazide moieties, Arch Pharm (Weinheim) 354 (2021). https://doi.org/10.1002/ardp.202100002.

[40] P.A. Teixeira de Moraes Gomes, M. Veríssimo de Oliveira Cardoso, I.R. dos Santos, F. Amaro de Sousa, J.M. da Conceição, V. Gouveia de Melo Silva, D. Duarte, R. Pereira, R. Oliveira, F. Nogueira, L.C. Alves, F.A. Brayner, A.C. da Silva Santos, V. Rêgo Alves Pereira, A.C. Lima Leite, Dual Parasiticidal Activities of Phthalimides: Synthesis and Biological Profile against Trypanosoma cruzi and Plasmodium falciparum, ChemMedChem 15 (2020) 2164–2175. https://doi.org/10.1002/cmdc.202000331.

[41] M.G. Da Silva, J.F. Cardoso, F.B. Perasoli, R.T. Branquinho, R.S. Mourão, H.D.S. Tavares, M.L.C.T. Xocaira, D.S.M. Guimarães, G.H.R. Viana, F.D.P. Varotti, G.R. Da Silva, Nanoemulsion composed of 10-(4,5-dihydrothiazol-2-yl)thio)decan-1-ol), a synthetic analog of 3-alkylpiridine marine alkaloid: development, characterization, and antimalarial activity, European Journal of Pharmaceutical Sciences 151 (2020) 105382. https://doi.org/10.1016/j.ejps.2020.105382.

[42] S.M. Divatia, D.P. Rajani, S.D. Rajani, H.D. Patel, Novel thiosemicarbazone derivatives containing benzimidazole moiety: Green synthesis and anti-malarial activity, Arabian Journal of Chemistry 12 (2019) 1641–1651. https://doi.org/10.1016/j.arabjc.2014.09.007.

[43] N.K. Nkungli, A.D.T. Fouegue, S.N. Tasheh, F.K. Bine, A.U. Hassan, J.N. Ghogomu, In silico investigation of falcipain-2 inhibition by hybrid benzimidazole-thiosemicarbazone antiplasmodial agents: A molecular docking, molecular dynamics simulation, and kinetics study, Mol Divers 28 (2024) 475–496. https://doi.org/10.1007/s11030-022-10594-3.

[44] R. Matsa, P. Makam, M. Kaushik, S.L. Hoti, T. Kannan, Thiosemicarbazone derivatives: Design, synthesis and in vitro antimalarial activity studies, European Journal of Pharmaceutical Sciences 137 (2019) 104986. https://doi.org/10.1016/j.ejps.2019.104986.
